# Expression of KID syndromic mutation Cx26S17F produces hyperactive hemichannels in supporting cells of the organ of Corti

**DOI:** 10.3389/fcell.2022.1071202

**Published:** 2023-01-09

**Authors:** Ana C. Abbott, Isaac E. García, Felipe Villanelo, Carolina Flores-Muñoz, Ricardo Ceriani, Jaime Maripillán, Joel Novoa-Molina, Cindel Figueroa-Cares, Tomas Pérez-Acle, Juan C. Sáez, Helmuth A. Sánchez, Agustín D. Martínez

**Affiliations:** ^1^ Centro Interdisciplinario de Neurociencias de Valparaíso, Instituto de Neurociencia, Facultad de Ciencias, Universidad de Valparaíso, Valparaíso, Chile; ^2^ Facultad de Medicina Veterinaria y Agronomía, Instituto de Ciencias Naturales, Universidad de las Américas, Viña del Mar, Chile; ^3^ Laboratorio de Fisiología Molecular y Biofísica, Facultad de Odontología, Universidad de Valparaíso, Valparaíso, Chile; ^4^ Centro de Investigaciones en Ciencias Odontológicas y Médicas, CICOM, Universidad de Valparaíso, Valparaíso, Chile; ^5^ Facultad de Ingeniería, Arquitectura y Diseño, Universidad San Sebastián, Santiago, Chile; ^6^ Computational Biology Lab, Centro Basal Ciencia & Vida, Universidad San Sebastián, Santiago, Chile; ^7^ Escuela de Química y Farmacia, Facultad de Farmacia, Universidad de Valparaíso, Valparaíso, Chile

**Keywords:** connexin, hemichannel, gap junction, syndromic deafness, cochlea, organ of Corti

## Abstract

Some mutations in gap junction protein Connexin 26 (Cx26) lead to syndromic deafness, where hearing impairment is associated with skin disease, like in Keratitis Ichthyosis Deafness (KID) syndrome. This condition has been linked to hyperactivity of connexin hemichannels but this has never been demonstrated in cochlear tissue. Moreover, some KID mutants, like Cx26S17F, form hyperactive HCs only when co-expressed with other wild-type connexins. In this work, we evaluated the functional consequences of expressing a KID syndromic mutation, Cx26S17F, in the transgenic mouse cochlea and whether co-expression of Cx26S17F and Cx30 leads to the formation of hyperactive HCs. Indeed, we found that cochlear explants from a constitutive knock-in Cx26S17F mouse or conditional *in vitro* cochlear expression of Cx26S17F produces hyperactive HCs in supporting cells of the organ of Corti. These conditions also produce loss of hair cells stereocilia. In supporting cells, we found high co-localization between Cx26S17F and Cx30. The functional properties of HCs formed in cells co-expressing Cx26S17F and Cx30 were also studied in oocytes and HeLa cells. Under the recording conditions used in this study Cx26S17F did not form functional HCs and GJCs, but cells co-expressing Cx26S17F and Cx30 present hyperactive HCs insensitive to HCs blockers, Ca^2+^ and La^3+^, resulting in more Ca^2+^ influx and cellular damage. Molecular dynamic analysis of putative heteromeric HC formed by Cx26S17F and Cx30 presents alterations in extracellular Ca^2+^ binding sites. These results support that in KID syndrome, hyperactive HCs are formed by the interaction between Cx26S17F and Cx30 in supporting cells probably causing damage to hair cells associated to deafness.

## 1 Introduction

Connexins (Cxs) are transmembrane proteins pivotal for cell communication as they assemble into hemichannels (HCs) and gap junction channels (GJCs). HCs connect the cell with its external milieu and are relevant pathways for paracrine or autocrine signaling, whereas GJCs connect the cytoplasm of cells in contact, allowing direct cell-cell interactions. In addition, both HCs and GJCs allow the passage of molecules and ions that are essential for maintaining cell homeostasis (Harris, 2007) (e.g., ATP, glucose, NAD^+^, and second messengers, among others) (Kang et al., 2008). Under normal conditions, extracellular Ca^2+^ and resting membrane potential keep HCs with low open probability (Bennett et al., 2016; Lopez et al., 2016), and depending on the Cx isoform, the open probability of HCs can be increased by diverse metabolic and signaling mechanism including, phosphorylation, nitrosylation, interaction with calcium-calmodulin and CO_2_ (Sáez et al., 2003; Nijjar et al., 2020).

The human genome presents 21 Cx genes with age and tissue-specific distribution (Willecke et al., 2002; Söhl and Willecke, 2003). For example, Cx26 and Cx30 are co-expressed in glia, skin, and cochlear supporting cells, among others (Ahmad et al., 2003; Forge et al., 2003; Zhao and Yu, 2006). In the membrane, Cxs form different oligomeric channels: homomeric, when HCs are composed of the same Cx type, and heteromeric, when different Cx isoforms conform the channel. The configuration adopted by HCs and a GJCs is extremely important because each Cx confers specific regulatory and intrinsic biophysical properties to the channels, including conductance, permeability, and voltage dependence (Koval et al., 2014).

Mutations in the Cx26 gene (*GJB2*) can produce genetic sensorineural hearing loss due to cochlear malfunction (Wingard and Zhao, 2015; Verselis, 2017). The exact role of Cxs in the functioning of the cochlea is under debate, but it has been suggested that it involves the regulation and recycling of K^+^, pH maintenance, and the passage of molecules like ATP and IP_3_ between supporting cells (Gossman and Zhao, 2008; Verselis, 2017), maintaining the sensitivity and viability of hair cells, and the general homeostasis of the auditory sensory epithelium (Wan et al., 2013). The most common non-syndromic deafness (DFNB1A) has been linked to more than 300 mutations in GJB2 (see http://deafnessvariationdatabase.org/letter/g, then select “GJB2″); these mutations can be missense, non-sense, frameshift, insertions, and deletions; of which non-sense/truncating mutations, such as 35delG, are the prevalent forms in some populations (Mammano, 2018). However, characterization of these mutants in exogenous expression systems have shown that they generally do not form GJCs or that GJCs formed present altered gap junctional permeability (Martínez et al., 2009; García et al., 2016b). Besides, a small group of mutations with a dominant inheritance pattern produces severe skin problems and correspond to syndromic deafness (Avshalumova et al., 2014; García et al., 2016a). An example of syndromic disease is the KID characterized by severe deafness and skin abnormalities such as palmoplantar keratoderma, erythrokeratodermia, ichthyosis, and in some cases, corneal keratitis that leads to blindness (Skinner et al., 1981; García et al., 2016a).

Most syndromic mutations in Cx26 are clustered in the amino-terminal domain (NT) and in the para-helix domain facing the internal and external vestibule of the channel pore, respectively (Sanchez and Verselis, 2014; García et al., 2016b) and in most cases, they form hyperactive HCs (Lee and White, 2009; Levit et al., 2012; Shuja et al., 2016) with altered gating properties (García et al., 2018b). Moreover, some KID mutants, like Cx26S17F, form hyperactive hemichannels only when co-expressed with other wild-type (WT) Cxs, like Cx43 (García et al., 2015). However, the presence of hyperactive HCs in the cochlea of a KID mouse model has not been determined yet.

In our previous studies, we showed that some KID mutations in the NT of Cx26 (e.g., S17F, G12R, and N14Y) produce aberrant Cx-Cx interactions that could lead to the formation of heteromeric HCs and GJCs with Cx43 (García et al., 2015). Since Cx26 and Cx43 are co-expressed in skin cells, we have proposed that the channels resulting from the oligomerization of mutant Cx26 and Cx43 are responsible for the skin phenotype observed in KID (García et al., 2016b). Moreover, keratinocytes and supporting cells of the organ of Corti also express Cx30 together with Cx26 (Zhao and Santos-Sacchi, 2000; Ahmad et al., 2003; Forge et al., 2003). In fact, Cx26 and Cx30 co-assemble into heteromeric channels in most cochlear supporting cells (Ahmad et al., 2003; Sun et al., 2005) with some heterogeneity due to differences in the expression ratio between Cx26 and Cx30, for example, Deiters’ cells express more Cx30 than Cx26 (Sun et al., 2005; Ortolano et al., 2008; Jagger and Forge, 2015; Liu et al., 2015). However, it remains unknown whether Cx26-KID mutants interact with Cx30 producing aberrant HCs and GJCs and whether such interaction alters the inner ear homeostasis.

In this study, we found that cochlear tissue expressing Cx26S17F presented increased membrane permeability in the supporting cells of the organ of Corti through the formation of hyperactive HCs, producing damage of hair cells in the organ of Corti. Moreover, we show that mutant Cx26S17F can associate with Cx30, producing hyperactive HCs with reduced sensitivity to extracellular Ca^2+^ and standard HC blockers. Molecular dynamic models of mutant heteromeric show alteration in residues involved in Ca^2+^ coordination. These results provide an explanation for the deafness phenotype in KID syndrome.

## 2 Materials and methods

*Generation of constitutive knock-in* (*KI*) *Cx26S17F.* Cx26S17F carrier mice (Cx26^+/floxS17F^) kindly donated by Dr. Klaus Willecke (Schütz et al., 2011) to Emma Infra Frontiers repository (strain EM:05215) was acquired to reproduce KID syndrome in mice. For this, Cx26^+/floxS17F^ mice, which has the Cx26 WT gene flanked by loxP sites and Cx26S17F cloned after the WT gene, allowed the expression of Cx26S17F under the WT promoter in every tissue that expressed Cx26, after crossbreeding with CMV-Cre mice (strain #6054, Jackson Laboratory), giving rise to KI Cx26S17F (Cx26^+/S17F^; KI) mice that presented skin problems as shown by skin disease (Supplementary Figure S1). Genotyping was done by PCR as described (Schütz et al., 2011) using the following primers loxP-F: CTA TCA GCA GCC TAG AGG AGG, and loxP-R: CAT GAT GCG GAA GAT GAA GAG, with an expected amplicon of 250 bp for Cx26 WT allele, 330 bp for floxed allele Cx26^+/floxS17F^ and 380 bp after deletion of Cx26 WT gene for KI Cx26S17F (Supplementary Figure S1B); and Cre F: GCG GTC TGG CAG TAA AAA CTA TC and Cre R: GTG AAA CAG CAT TGC TGT CAC TT, with an expected amplicon of 100 bp (not shown), according to the manufacturer’s instructions.

*Cochlear explant cultures.* Cochleae were obtained according to the protocols approved by the Ethical and Animal Care Committee of the Universidad de Valparaíso, considering all efforts to avoid mice suffering and the use of *in vitro* approaches when available. Briefly, P1 to P7 animals (7 days postnatal) were sacrificed by decapitation. The explants were obtained by skull dissection from the temporal bone; both inner ears located in the temporal bone were isolated, and cochleae were extracted as described (Parker et al., 2010). Then, once separated from the spiral ligament, the sensory epithelium was transferred on glass coverslips pre-coated with Geltrex (Gibco) in a plating medium (Dulbecco’s Modified Eagle Medium, supplemented with 5% FBS and 5% HS), and maintained for 24 h at 37°C in a humified incubator under 5% CO_2_ atmosphere and 95% humidity (Supplementary Figure S2).

*Ex-vivo Cx26S17F conditional KI* (*cKI Cx26S17F*)*.* Cx26^+/floxS17F^ cochlear explants were treated with Cre-recombinase recombinant protein fused to a TAT sequence (TAT-Cre) enzyme, allowing the recombination to express cKI Cx26S17F *ex-vivo.* TAT-Cre recombinase was used at 10 U/ml in culture media and incubated overnight at 37°C, according to the manufacturer’s instructions (Sigma-Aldrich) (Supplementary Figure S3). All experiments were done after ∼16 h incubation.

*Cell culture and transfections.* HeLa cell culture and transfections were done as described (García et al., 2015). Cx26S17F was cloned in pcDNA3.1 C T-GFP-TOPO (Invitrogen). A plasmid with the Cx30 cloned in a vector containing msfGFP was acquired from the Addgene reservoir (#69019; donated by Dr. David C. Spray. Albert Einstein College of Medicine, New York, USA) and subsequently cleaved to clone into the pNT-mCherry vector (Clonetech, kindly donated by Dr. María Estela Andrés, Universidad Católica de Chile), which allowed the visualization of Cx30 in two colors, according to the experiment to be carried out. For transient expression of the different Cxs in HeLa cells, transfections were performed using the protocol described for Lipofectamine 2000 (Invitrogen). For double transfections with Cx26S17F/Cx30, 1 µg of Cx26S17F and 0.7 µg of Cx30 plasmids were used. All the experiments were performed 16 h after transfection.

*Immunofluorescence and colocalization analysis.* Cochlear Explants were fixed in PBS-4% PFA for 1 h at room temperature, washed with PBS, and stored at 4°C until use. The following primary and secondary antibodies were used: mouse αCx26 and rabbit αCx30 (Lifetech); αMouse-Cy2 (Jackson InmunoResearch) and αRabbit-Cy3 (Jackson InmunoResearch). Antibodies were diluted in blocking solution (PBS-Triton X-100 1% + 2% BSA +2% normal goat serum) as follows: mouse αCx26 1:100; rabbit αCx30 1:100; αMouse-Cy2 1:500, αRabbit-Cy3 1:500. Additionally, the stereocilia were detected by phalloidin-Texas Red (Sigma-Aldrich) staining. In all the above experiments, the cells were examined and acquired in an upright confocal laser-scanning Nikon Eclipse C1-Plus microscope (Nikon Instruments) using the EZ-C1 software and were analyzed with the FIJI software (NIH). In addition, optical sections were acquired for every 1 µm in the z-axis, and 3D confocal images were reconstituted from confocal stack images using NIS elements software (Nikon Instruments). In addition, some experiments for stereocilia visualization were analyzed by super-resolution microscopy (Zeiss ELYRA S1, SR-SIM) at the Center for Advance Microscopy at the Universidad de Concepción, Chile (https://cmabiobio.cl/en/).

Immunofluorescence was carried out in transfected HeLa cells as described previously (García et al., 2015); with the following primary and secondary antibodies, respectively: mouse αCx26 1:200 (Lifetech), rabbit αCx30 (Lifetech), and αMouse-Cy2 1:1000 (Jackson InmunoResearch) and αRabbit-Cy3 1:1000 (Jackson InmunoResearch). Cell nuclei were counterstained with DAPI at room temperature for 10 min. Expression of each Cx was quantified in each optical section of 1 µm of confocal stack images using NIS elements software (Nikon Instruments). Plasma membrane was not labeled directly, instead we estimated its localization between cellular appositions (cell-cell contacts) and cell surface, observed using the bright field light from confocal microscope images, similar criteria has been used before (Lauf et al., 2002).

To evaluate the colocalization strength of Cx30 with Cx26WT or Cx26S17F, Pearson and Manders’s coefficients were calculated by cellular area in images showing HeLa cells co-expressed Cxs, using NIS elements software (Nikon Instruments). Ten ROIs of the same size were randomly distributed in a 1 µm optical section in the z-axis for each cellular compartment, membrane, cytoplasm, and the cellular appositional zone (GJ Zone). ROIs that did not show expression of both Cxs were not considered in this quantification. The threshold of every image was set visually. Statistical analyzes were made in Graph Pad Prism software (GraphPad Software Inc., USA).

*Proximity ligation assay* (*PLA*)*.* PLA methodology was used according to the manufacturer’s instructions (Duolink, Sigma-Aldrich). The PLA anti-rabbit minus probe binds to the αCx30 antibody, whereas the PLA anti-mouse plus probe binds to the antibody against αCx26 or with the probable interaction partner respectively. Subsequently, the samples were incubated with secondary antibodies conjugated with the PLA probe. After that, amplification and detection protocols were done as manufacturer´s instructions. In all the above experiments, the cells were examined and acquired in an upright confocal laser-scanning Nikon Eclipse C1-Plus microscope (Nikon Instruments) using the EZ-C1 software and were analyzed with the FIJI software (NIH).

*Fluorescence recovery after photobleaching* (*FRAP*) *assessed the functional state of gap junctions.* Cochlear explants or HeLa cells were loaded with 1.6 µM calcein-AM (MW 994.86; net charge -4, Sigma-Aldrich) dissolved in Hanks for 30 min at 37°C protected from light. Then, the excess calcein was removed with two washes of 5 min with Hanks. Using 100% laser power of the confocal microscope, the fluorescence of one of the coupled cells through GJs was bleached. Subsequently, using the 5% power laser, pictures were taken every 1 min for 10 min, obtaining the fluorescence recovery rate in the bleached cell and the concomitant fluorescent reduction in the coupled contacting cells. Instead of bleaching 1 cell for cochlear explants, we bleached a small group of supporting cells (2-4) using 100% laser power. This was because cells in the explants were smaller and more packed between them; in addition, the organ of Corti has two three-layered cells around the tunnel of Corti, making it challenging to bleach just 1 cell. After bleaching, the fluorescent recovery rate was measured in different supporting cells by taking pictures every 1 min for 10 min using 5% laser power. FRAP experiments were performed and acquired in an upright confocal laser-scanning Nikon Eclipse C1-Plus microscope (Nikon Instruments) using the EZ-C1 software and were analyzed with the FIJI software (NIH).

*Dye uptake measurements.* The dye uptake approach assessed the functional state of HCs expressed in cochlear explants or HeLa cells (García et al., 2015). Dye uptake was determined in cochlear explants maintained for 24 h on coverslips 25 mm in diameter. The culture medium was removed and changed to normal Hank´s solution (mM: 10 HEPES, 140 NaCl, 5.3 KCl, 0.1% glucose, 0.34 Na_2_HPO_4_, 1.26 CaCl_2_, 0.5 MgCl_2_; pH 7.4) or in Hank´s divalent cation-free solution (DCFS) containing DAPI (1 µM) as HC activity tracer. Fluorescence microscope pictures were taken in time-lapse mode every 30 s for 10 min in the basal condition (with extracellular Ca^2+^), 5 min in DCFS, and then 5 min in the presence of DCFS with the 100 µM carbenoxolone (CBX). At the beginning and end of the recording, phase contrast photographs were taken to assess cell morphology.

Transiently or stable transfected HeLa cells were grown on 25 mm diameter coverslips. Experiments were done 16 h after transfection in normal Hank´s recording solution and DCFS. Both solutions contained 5 µM ethidium bromide (Etd) as an HCs activity tracer. Pictures were taken every min for 20 min in the basal condition, 10 min in DCFS, and then 10 min in DCFS plus 100 µM La^3+^. To assess cell morphology, contrast photographs were taken at the beginning and end of the recording. All dye uptake experiments were performed in an upright Nikon Eclipse TE 2000U microscope (Nikon Instruments) using the NIS Elements software and were analyzed with the FIJI software (NIH).

*Connexin hemichannel currents.* Molecular biology, channel expression, and electrophysiology in *Xenopus laevis* oocytes were performed as described (García et al., 2015; 2018b). cDNA for hCx30, hCx26, or hCx26S17F was subcloned in the pGEM-HA vector (Promega) for *in vitro* transcription. Macroscopic currents were evaluated at 24 or 48 h post cRNA injection in oocytes bathed in extracellular solution containing 1.8 mM Ca^2+^. All experiments were performed at room temperature (RT, 22°C). Macroscopic Cx HCs currents were recorded using the two-electrode voltage-clamp technique using a Warner oocyte clamp (OC-725C; Warner Instruments). Micropipettes housing the two Ag/AgCl electrodes were pulled to a resistance of 0.5–1 MΩ and filled with 3 M KCl. The bath solutions (mM: 118 NaCl, 1,8 CaCl_2_, 2 KCl, and five HEPES, pH 7.4) was connected to two 3 M KCl pools using agar salt bridges. Membrane currents were recorded from oocytes in response to depolarizing voltage steps from a holding potential of −80 mV and stepped in 20 mV increments from–60 mV to 60 mV and returning to −80 mV. Currents were low-pass filtered at 200 Hz and sampled and digitized at 2 kHz. Current amplitude was determined as *I*_*tail*_ *(V)/I*_max_ for each voltage tested.

*Cell damage/death quantification.* Cell damage/death was quantified in HeLa cells with the Annexin V/Propidium iodide (PI) assay (BioLegend^®^), according to the manufacturer’s instructions with modifications. Cells were seeded on 25 mm coverslips, washed twice with PBS and incubated with 400 µl Annexin V binding buffer, plus 5 µl of Pacific Blue™ Annexin V and 10 µl of PI solution. Solutions were homogenized, and cells were incubated for 15 min at RT. Then, samples were washed twice with PBS and fixed with 4% PFA for 30 min at RT. After fixation, cells were washed and mounted with anti-fade mounting medium Fluoromount-G (Sigma-Aldrich). All the experiments were kept in dark till assessment. The cells were examined and acquired in an upright confocal laser-scanning Nikon Eclipse C1-Plus microscope (Nikon Instruments) using the EZ-C1 software and were analyzed with the FIJI software (NIH). Cx30 in this experiment was tagged with mCherry (Ex/Em λ= 587/610), which shares similar spectrum with PI (Ex/Em λ= 535/617). The difference between Cx30-mCherry and PI was assessed by morphology, since PI stains the nuclei and cytoplasm with fade red in healthy cells and strong fluorescent red in necreotic cells, which are also round.

*Intracellular Ca* ^*2+*^ *signal measurement.* Basal intracellular Ca^2+^ signal was evaluated in HeLa Cells using Fura-2 (Invitrogen). Cells seeded on 25 mm coverslips were loaded with 5 µM Fura-2 diluted in serum-free medium for 30 min at 37°C and washed two times with Hank´s solution. Coverslips were placed in the recording chamber filled with 1 ml of Hank´s solution. The imaging protocol involved data acquisition of light emission at 510 nm due to excitation at 340 nm and 380 nm. The ratio was obtained by dividing the emission fluorescence image value at 340-nm by the 380 nm excitation on a pixel-by-pixel base (Ca^2+^ signal = F340 nm/F380 nm). Extracellular [Ca^2+^]_e_ was changed every 3 min from nominal Ca^2+^-free solution to 10 μM, 100 μM, 500 μM, 1 mM, 1.5 mM Ca^2+^ influx at different [Ca^2+^]_e_ was estimated from changes in [Ca^2+^]_i_ rate of increments in every [Ca^2+^]_e_. Experiments were performed in an upright Nikon Eclipse TE 2000U microscope (Nikon Instruments) using the NIS Elements software and were analyzed with the FIJI software (NIH).

*Molecular dynamics.* The Cx26WT/Cx30 and Cx26S17F/Cx30 putative heteromeric HC models were constructed using the coordinates of crystallographic structure from the cryo-EM structure of Cx50 (PDB code: 7JJP; (Flores et al., 2020); as a template. This structure has a resolution of 1.9 Å, allowing us to accurately model the sidechain of residues. Missing residues and sections from original coordinates were modeled using MODELER (Martí-Renom et al., 2000). The stoichiometry used was Cx26:Cx26:Cx30:Cx30:Cx26:Cx30 because it is the most abundant composition found *in vitro* (Naulin et al., 2020). The best models were chosen using the MAIDEN program (Postic et al., 2015). Each HCs were inserted into a 1-palmitoyl-2-oleoylsn-glycero-3-phosphocholine (POPC) lipid membrane considering the spatial arrangements of the protein with respect to the hydrocarbon core of the lipid bilayer, as obtained from the OPM database (Lomize et al., 2006). A 150 × 150 × 110Å box, including the protein, lipids, TIP3 water molecules, and 150 mM KCl, was generated using the INFLATEGRO technique (Kandt et al., 2007). The CHARMM-22 and the CHARMM-36 force fields (MacKerell et al., 1998) were used for protein and lipids, respectively. Initial force constraints were used in NVT dynamics, but these were gradually reduced. The system was then equilibrated for 50 ns at 310 K using NPT dynamics without any constraints. Three independent MD simulations of 20 ns were performed by starting the simulation from different seeds, using NPT dynamics for data collecting and statistical analysis with set points of 1 atm and 310 K using the Nose–Hoover thermostat and Parrinello-Rahman barostat. All simulations were performed using Gromacs 2019 (Abraham et al., 2015). The PME method was used for full long-range electrostatics within a relative tolerance of 1 x 10-6. A cutoff distance of 12Å was applied to real-space Ewald interactions, with a smooth switching function applied between 10 and 12Å to account for Van der Waals (VdW) interactions. The SHAKE algorithm was applied to constrain bond lengths to all hydrogen atoms. All the simulations were performed on the infrastructure of the Computational Biology Laboratory at Fundación Ciencia & Vida (Santiago, Chile).

*Study design and statistical analysis*. Sample sizes were not statistically pre-determined, but they were estimated based on previous studies. Considering that *in vitro* experiments were used in transfected cells, they can be considered technical replicates. Despite that, experiments were performed in four or more different independent cultures for every method, and more than four coverslips per day of the experimental condition were considered. Every double-transfected cell in the microscopy field of view was analyzed. For cochlear explants, only two animals were used per condition, and 120 ROIs positioned around the nuclei area were analyzed in different cell types. The researcher was not blinded during the experiments, but the experiments were analyzed randomly.

Results are expressed as means ± SEM, for most results data were normally distributed according to the Kolmogorov-Smirnov test. In some experiments, outliers were removed after identification using the ROUT Method (GraphPad Software Inc, La Jolla, CA, United States) using Q = 1%. Statistical comparisons were performed using a two-tailed t-test comparison between groups, and results were expressed in the graphs as *p < 0.05; **p < 0.005, ***p < 0.001; ****p < 0.0001 accordingly. When data were not distributed normally, a non-parametric t-test was used (Mann-Whitney test). Statistical analysis for multiple comparisons was corrected by the Bonferroni-Dunn method. All data analyses of every experiment were done in Excel (Microsoft Inc.) and GraphPad Prism 6 software (GraphPad Software Inc.).

## 3 Results

### 3.1 Expression of Cx26S17F changes the subcellular distribution of connexins in supporting cells of the organ of Corti

In order to understand the outcome of Cx26S17F expression in the inner ear, we used cochlear explants from WT and transgenic mouse Cx26^+/floxS17F^, a mice strain that carries Cx26S17F mutation downstream to Cx26 gene flanked by loxP sequences and does not show evident abnormal phenotype (Schütz et al., 2011). Furthermore, the F1 progeny obtained from crossing a female Cx26^+/floxS17F^ with a male CMV-Cre mouse (ubiquitous Cre recombinase activity) expressed Cx26S17F instead of Cx26 (KI Cx26S17F) exhibit skin damage, resulting in a suitable model for KID syndrome (Supplementary Figure S1). In addition, the macroscopic organization of the cochlea and vestibular systems extracted from KI S17F mouse seems to be similar to WT cochleae (Supplementary Figure S2), suggesting that expression of Cx26S17F did not alter cochlear development. However, we found a high incidence of death at P2-P3 in KI Cx26S17F mice, an age at which the mouse’s inner ear is still immature. Therefore, we used another strategy in which the expression of conditional Cx26S17F *knock-in* (cKI Cx26S17F) was induced by the treatment of cochlear explants from Cx26^+/floxS17F^ animals with TAT-Cre recombinase, which is a recombinant cell-permeant fusion Cre-recombinase protein, that allows the diffusion of Cre enzyme into the cells for *in situ* and *ex-vivo* recombination (Kang et al., 2018) (Supplementary Figure S3).

After 1 day *in vitro* (1 DIV), control cochlear explants showed normal cellular integrity and organization (Supplementary Figures S3A, B). Using higher magnification under brightfield and DAPI staining of the nuclei, the following cell types were recognized: ISC, inner supporting cells; IHC, inner hair cells; PC, pillar cells; OHC, outer hair cells; OSC, outer supporting cells; and F, fibrocytes. Since the stereocilium is a very sensitive structure that might break down after mechanical stress, we analyzed the integrity of cochlear cells stained with fluorescent phalloidin. Phalloidin-stained cochlear explants showed normal cellular morphology, including perfectly aligned stereocilia (Supplementary Figure S3B). In addition, treatment of WT cochleae with a concentration of 10 U/ml of TAT-Cre for 24–30 h did not alter the integrity of stereocilia, however treatment with 100 U/ml did produce disorganization of OHC stereocilia (Supplementary figure S4 **),** suggesting that treatment with higher concentrations of TAT-Cre can deteriorate cochlear explants. Therefore, through the study we use 10 U/ml of TAT-Cre for our experiments. Finally, as a control of the TAT-Cre strategy, cochlear explants from Ai9 mice (Jackson #007909; kindly donated by Dr. Chiayu Chiu, Universidad de Valparaiso, Chile) were used to demonstrate somatic recombination *ex-vivo*, since these animals express tdTomato after recombination. Partial expression of tdTomato was clearly observed after 16 h of incubation with TAT-Cre (Supplementary Figure S5).

**FIGURE 1:**
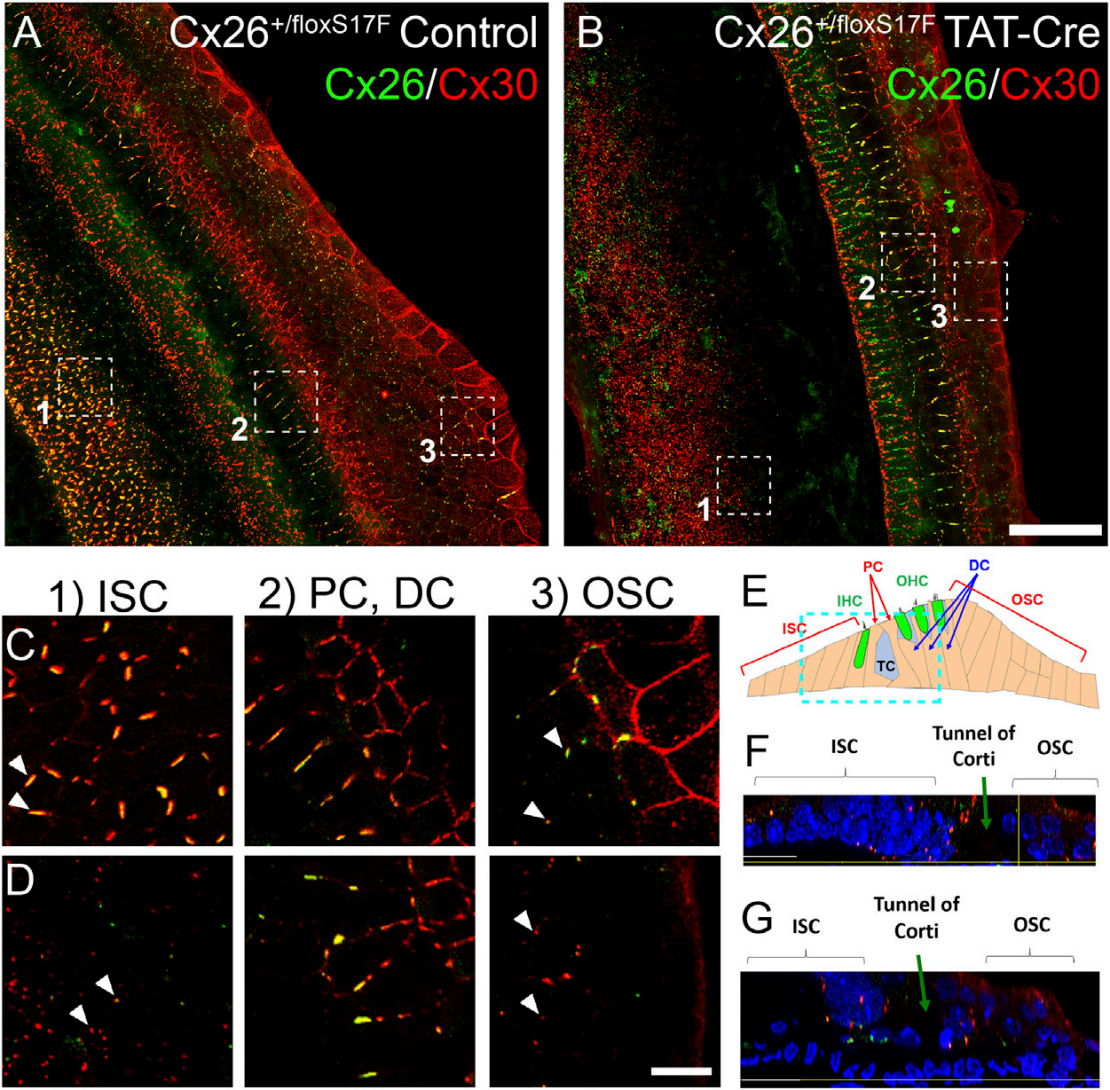
Expression of Cx26S17F changed the distribution of Cx26 and Cx30 in supporting cells of the organ of Corti in cochlear explants. a, **(C)**. Control cochlea (Cx26^+/floxS17F^, **(A)** showed the typical distribution of Cx26 (green) and Cx30 (red), forming heteromeric GJ plaques, as seen in the magnified views of 1 μm thick of OSC, PC, and DC and ISC **(C)**. b, **(D).** Expression of cKI Cx26S17F induced by the addition of TAT-Cre (Cx26^+/floxS17F^ TAT-Cre, **(B)** changed the distribution of mutant Cx26S17F and Cx30. Magnified views are shown in **(D)**, where GJ plaques are reduced, and both Cxs are retained in the intracellular space in OSC. In addition, the ISC expression of GJCs is totally lost. PC and DC did not show any differences. **(E).** Diagram of the organ of Corti, showing the relative position of the focal plane used for **(F)** and **(G)**, respectively (cyan rectangle). **(F)**, **(G).** Transversal views of representative zones of the cultured organ of Corti [from **(A)** and **(B)**, respectively]. Scale bars: 50 μm **(B)**, 5 μm **(D)**. Supplementary Figure 6 shows the same images of Cx26 and Cx30 localization in separate color panels.

The subcellular localization of Cxs induced by the expression of Cx26S17F in explants from Cx26^+/floxS17F^ mice incubated with (cKI Cx26S17F) or without (control) TAT-Cre enzyme was carried out by immunofluorescence. In control explants, strong co-localization of Cx26 (green) and Cx30 (red) was detected in supporting cells of the organ of Corti (Figure 1). Closer views (Figure 1C) of OSC revealed Cx26 and Cx30 co-localization in our culture conditions, forming large GJ plaques. The PC, which forms the tunnel of Corti, and Deiters cells, also showed GJ plaques bigger than those observed in OSC but were mainly formed by Cx30 (Figure 1C). In contrast, ISC showed massive amounts of smaller GJ plaques (Figure 1C). An orthogonal view of control explants showed that Cx distribution was mainly present in the lower and upper parts of supporting cells (Figure 1F). Finally, we cannot exclude the possibility that at the edges of OSC some fibrocytes can be present in the culture because part of the lateral wall can remain attached to the organ of Corti, which also express Cx26 and Cx30 (Furness, 2019).

Several changes were observed in cochlear explants from Cx26^+/floxS17F^ treated with TAT-Cre (cKI Cx26S17F; Figures 1B,D). The cells of OSC and ISC showed Cx26S17F and Cx30 that mostly co-localized in the intracellular space and a few small GJC plaques (Figure 1D). However, we did not observe changes in Cx expression in pillar and Deiters cells. An orthogonal view of the cKI Cx26S17F organ of Corti revealed that Cx distribution was altered and affected the nuclear morphology of supporting cells, which were smaller and more compact than in control cells (Figure 1G).

### 3.2 Cx26S17F forms hyperactive hemichannels in supporting cells of the organ of Corti and causes loss of stereocilia integrity

**FIGURE 2:**
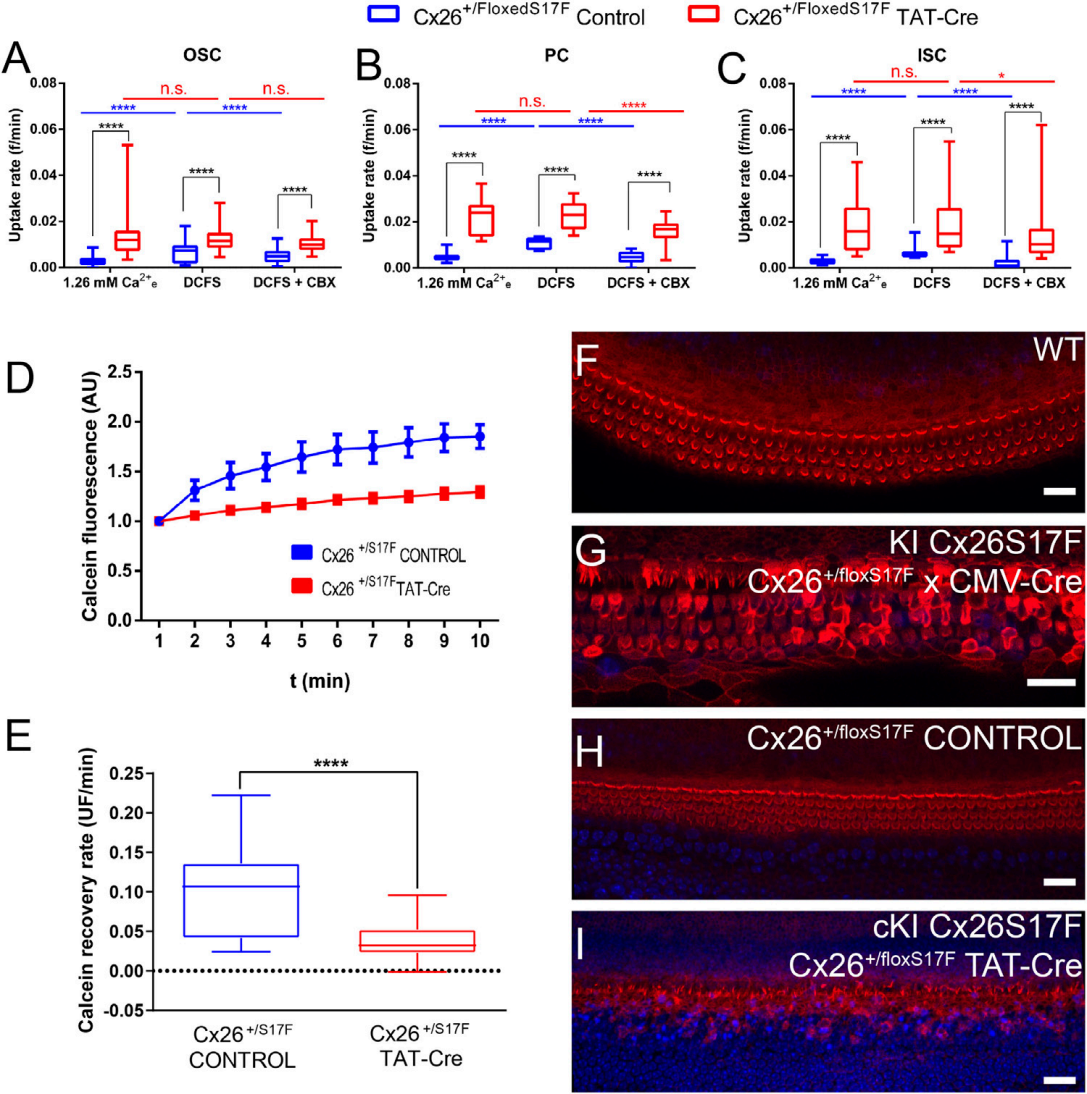
cKI Cx26S17F expression increased the activity of HCs in supporting cells, reduced intercellular GJCs coupling, and affected the morphology of hair cell stereocilia in cochlear explants. **(A–C)**. Rate of DAPI uptake by OSC, PC, and ISC in control (blue) and TAT-Cre (red) treated cochlear explants of Cx26^+/floxS17F^ mice, shown as box and whiskers plots showing the minimum and maximum values, and the box represents the 25th and 75th percentiles, and the central line marks the median. Intragroup comparisons are presented in blue (control) and red (TAT-Cre). **(D)** Representative fluorescence recoveries in FRAP experiments show a severe reduction of intercellular communication between supporting cells of cKI Cx26S17F cochlear explants (red) compared to the control (blue). **(E)** Average calcein recovery rates of experiments are shown in d. **(F–I)**. Representative views of stereocilia in the organ of Corti obtained from cochlear culture from the different conditions, phalloidin stained conjugated with Texas-Red (red, cytoskeleton) and DAPI (blue, nuclei). Statistical analysis, *p < 0.05; ****p < 0.0001 **(A–E)**.

The HCs function was evaluated by DAPI uptake in control (Cx26^+/floxS17F^) and cKI Cx26S17F (Cx26^+/floxS17F^ TAT-Cre) cochlear explants (Figures 2A–C). In control explants, OSC, PC, and ISC (Figures 2A–C) showed low DAPI uptake under basal conditions (1.26 mM [Ca^2+^]_e_), which then increased upon exposure to DCFS. Then, explants were treated with the HC blocker CBX, which reduced the uptake rate of DAPI in these cells. In contrast, in cochlear explants treated with TAT-Cre dye uptake rate in basal conditions was higher in OSC, PC, and ISC compared to control explants. The higher DAPI uptake rate observed in cKI Cx26S17F did not change after the basal recording solution was replaced by DCFS or in explants treated with CBX (Figures 2A–C). These results lead to two important observations: 1) In control explants, Cx HCs are present in supporting cells since the activity recorded was blocked by CBX and high [Ca^2+^]_e_; and 2) the expression of cKI Cx26S17F in cochlear explants induces the formation of hyperactive HCs, possibly by leading to the generation of aberrant heteromeric HCs containing mutant Cx26 and Cx30, with reduced sensitivity to extracellular Ca^2+^.

The functional state of GJCs in control and TAT-Cre treated Cx26^+/floxS17F^ cochleae was assessed by using the FRAP technique, we studied the intercellular diffusion of calcein (623 Da; net charge of z = -4; medial axial diameter of 6.5 Å) between neighboring supporting cells (Figures 2D, E). The reappearance of calcein over time was quantified in the bleached cell, and the slope was used to measure the functional state of GJCs. Representative traces showed that in control explants, calcein fluorescence recovered after 3 min, indicating the presence of functional GJCs whereas in *ex-vivo* cKI Cx26S17F fluorescence recovery was significantly reduced (Figure 2D), indicating that expression of Cx26S17F produce less or non-functional GJCs. Accordingly, recovery rates of bleached cells from cKI Cx26S17F explants were significantly lower than in control explants (Figure 2E).

Finally, we evaluated the general state of the hair cells in cochlear explants by staining stereocilia with phalloidin in WT, Cx26^+/floxS17F^, KI Cx26S17F, and cKI Cx26S17F. Cochlear explants from P7 WT mouse showed V-shaped stereocilia in the OHC and a more horizontal shape in IHC stereocilia bundles (Figure 2F), typical morphology described previously for WT animals (Ashmore, 2008). In contrast, KI Cx26S17F showed an altered phenotype in hair cell stereocilia, disrupted hair cell stereocilia, and even some stereocilia were lost, especially in the IHC (Figure 2G). Hair cell stereocilia in the carrier mouse Cx26^+/floxS17F^ did not show a marked difference compared to WT explants (Figure 2H). As expected, Cx26^+/floxS17F^ cochlear explants treated with 10 U/ml of TAT-Cre for the *ex-vivo* expression of Cx26S17F (cKI Cx26S17F) showed loss of stereocilia (Figure 2I), which was less dramatic than in KI Cx26S17F F1 mice. All these observations suggest that, even though hair cells did not express Cxs, the expression Cx26S17F is detrimental to the sensory epithelium of the inner ear.

### 3.3 Syndromic deafness mutant Cx26S17F interacts with Cx30

**FIGURE 3:**
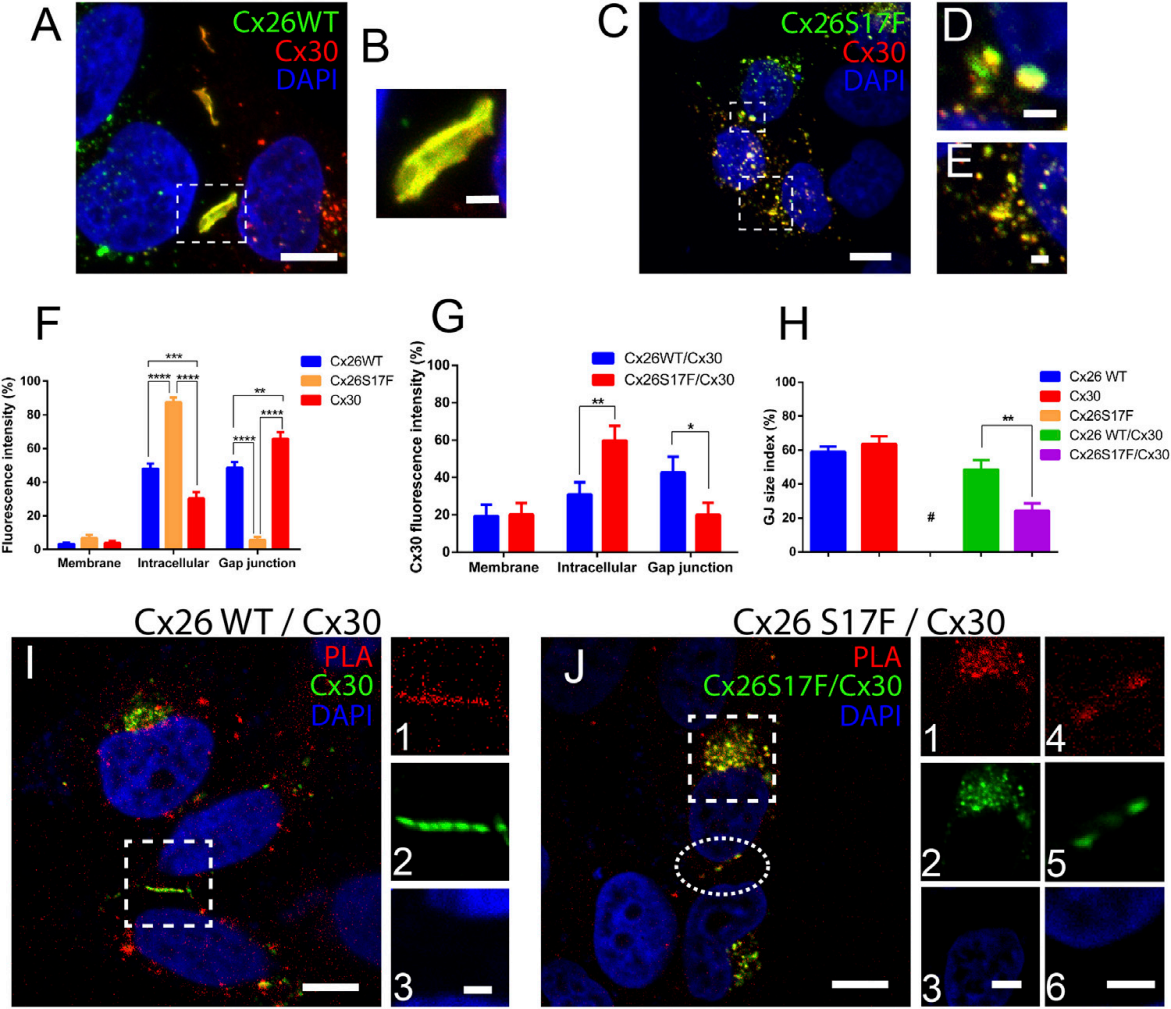
Mutant Cx26S17F co-localizes with Cx30 and changes the distribution of heteromeric HCs and GJCs in HeLa cells. **(A)**. Strong co-localization of Cx26WT (green) and Cx30 (red) in GJ plaques. **(B)**. Zoom of GJ plaques observed in **(A)**. **(C)**. Strong co-localization of Cx26S17F/Cx30 was observed mainly in intracellular compartments and occasionally in a few small plaques in the appositional zone. **(D,E)** Zoom of small GJ plaques **(D)** and intracellular **(E)** co-localization between Cx26S17F/Cx30. **(F,G)** Relative expression of each Cx by subcellular area in **(F)** cells expressing only Cx26WT, Cx26S17F, or Cx30 (homomeric configuration) and **(G)** cells co-expressing Cx26WT with Cx30 (blue) or Cx26S17F (red). **(H)**. GJ size index between neighbor cells expressing one Cx type or co-expressing Cx26WT with Cx30 or Cx26S17F. Mean values, SEM, and *p* values are indicated in Table 8. **(I, J)**. PLA (red) shows an association between Cx30 (green) with Cx26WT (no tag) **(I)**, and Cx26S17F **(J)**. Highlighted zone in **(I)** is shown in lateral panels 1) PLA, 2) Cx30, and 3) DAPI. Scale bar: 10 µm. Intracellular signal in the highlighted square in **(J)** is shown in 1) PLA, 2) Cx26S17F/Cx30 and 3) DAPI; and small GJCs are shown in a highlighted ellipse in 4) PLA 5) Cx26S17F/Cx30 and 6) DAPI. Scale bar: 3 µm. Scale bar: 10 µm **(A,C,I,J)**, 4 µm (j3 and 6) and 2 µm (b, d, e and i3). Statistical analysis, *p < 0.05; **p < 0.005, ***p < 0.001; ****p < 0.0001 **(H)**. N = 7. Supplementary Figure S7 shows the same images of Cx26 and Cx30 localization in separate color panels and Supplementary Figure 8 show positive and negative controls of PLA experiments.

As mention before, Cx26S17F may not produce functional HCs when expressed alone, hence the increased dye uptake observed in cochlear explants from cKI Cx26S17F can be the result of the formation of heteromeric HCs with co-expressed Cx30 in supporting cells. We explore this possibility in HeLa cells to see whether Cx26S17F and Cx30 can interact to form aberrant HCs. First of all, we performed immunocytochemistry and colocalization analyses in HeLa cells co-transfected with Cx26S17F and Cx30. We found Cx26 wild-type (Cx26WT) co-localized with Cx30 at cell membrane appositions forming large GJC plaques (Figures 3A, B; Supplementary Tables S1–3). By contrast, few fluorescence spots were located at appositional zones forming small GJC plaques between cells co-expressing Cx26S17F and Cx30 (Figures 3C,D). Most fluorescent signals were at intracellular compartments or non-appositional plasma membranes, suggesting that Cx26S17F retains Cx30 at intracellular compartments (Figures 3C–E).

To confirm that Cx26S17F affects the subcellular localization of Cx30, we quantified the fluorescence intensity in all cellular areas and measured the GJC plaque size index at appositional zones (see Methods). In cells transfected with Cx30 (Figure 3F), expression was found in the appositional zone forming large GJC plaques (∼65% of total, Figure 3H); approximately 30% was located in the intracellular space, and only a small fraction (<5%) remained in the non-appositional cell membrane. In cells transfected with Cx26WT, the protein was equally distributed between the intracellular space and GJC plaques (∼48% in each localization), with less than 10% localized at the non-appositional membrane. As previously reported (García et al., 2015), we found that homomeric Cx26S17F was located mainly in the intracellular space (>85%), suggesting that this mutation impaired the traffic of Cxs to the plasma membrane (Figure 3F and Supplementary Table S4). In double transfection cultures, Cx26WT and Cx30 (Figure 3G) showed a distribution like that observed for the respective homomeric WT channels (∼40%; Supplementary Table S5). Incidence of large GJC plaques at appositional zones and ∼20% of fluorescence spots were present at the non-appositional membrane. In contrast, Cx26S17F/Cx30 aggregates in cells co-expressing remained mainly in the intracellular space or non-appositional membrane zones (Figure 3G; Supplementary Table S6). In fact, Cx26S17F sequestered and retained ∼60% of Cx30 in the intracellular space and reduced the signal of Cx30 in the appositional zone to less than 20% (Figure 3G; Supplementary Table S7). The GJC size index for GJ plaques formed by Cx26S17F/Cx30 was significantly smaller than those formed by Cx26WT/Cx30 (Figure 3H; Supplementary Table S8). However, in cells co-expressing Cx26S17F/Cx30 ∼20% of the heteromeric fluorescent signal was located in the non-appositional membrane (Figure 3G).

The morphometric analyses of subcellular localization support the hypothesis that mutant Cx26S17F interacts with Cx30. To check this possibility more directly, we performed a PLA in cells co-expressing Cx26WT or Cx26S17F with Cx30. A strong signal of interaction was found either in cells co-expressing Cx26WT and Cx30 or Cx26S17F and Cx30, which suggest the formation of heteromeric HCs in non-appositional and appositional zones (Figures 3I, J). These results also indicate that mutation p.S17F in Cx26 did not change the property of Cx26 to interact with Cx30 and possible form heteromeric HCs. Two type of negative controls were perform for these study, first the PLA essay was done in non-transfected HeLa parental cell, and a second assay was done in HeLa cells co-transfected with Cx26 and Cx43, because Cx43 is not compatible to form heteromeric channels with Cx26 (Gemel et al., 2004) both control experiments show negative PLA signal (Supplementary Figure S8). A positive control was done in cells expressing only Cx26 but using two different primary antibodies against Cx26, a mouse monoclonal antibody and a rabbit polyclonal antibody, which recognizes different regions of the protein, this positive control show strong signal at GJ plaques and in intracellular compartments (Supplementary Figure S8).

### 3.4 Co-expression of Cx26S17F and Cx30 strongly reduces intercellular gap junctional communication mediated by Cx30

**FIGURE 4:**
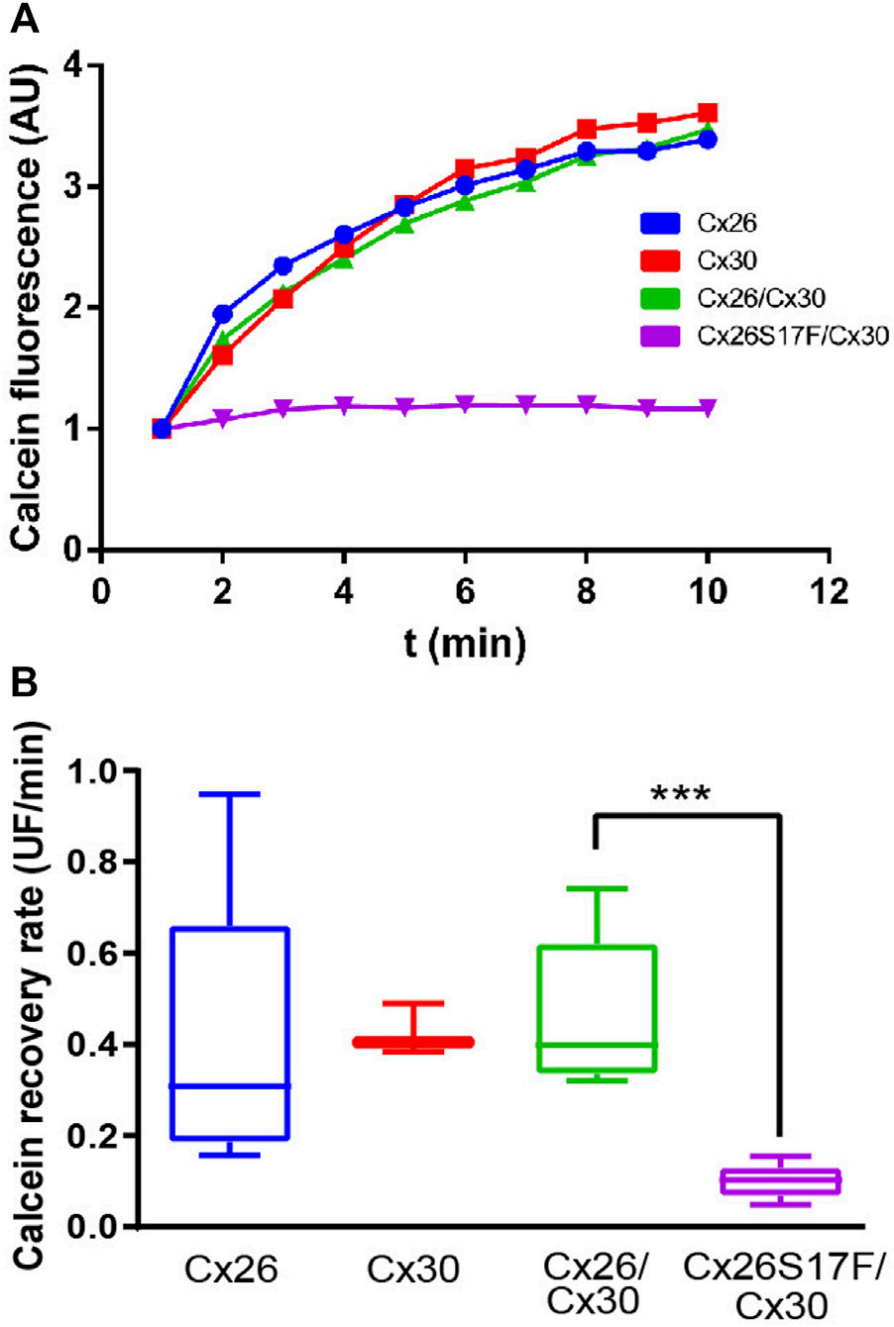
Expression of Cx26S17F impairs GJ functionality of Cx30. **(A, B)**. Co-expression of Cx26S17F/Cx30 strongly reduces gap junctional communication. **(A)** Representative fluorescence recovery of the bleached cell over time in FRAP experiments. **(B)** Quantification of total recovery rate. ***p < 0.001. Cx26 WT n = 2; Cx30 n = 2; Cx26WT/Cx30 n = 4; Cx26S17F n = 4.

Since the mutant Cx26S17F exerts a trans-functional dominant negative effect over Cx43 GJCs (García et al., 2015), we decided to study whether it exerted a similar effect over Cx30 GJCs. Using FRAP analysis in cells expressing homotypic/homomeric GJCs formed by Cx26WT and/or Cx30 revealed a similar fluorescent recovery rate of calcein (Figures 4A, B), consistent with the formation of functional GJCs by both Cxs. In addition, cells co-transfected with both Cxs WT present similar fluorescent recovery rate, suggesting that the interaction of Cx26 with Cx30 produce functional GJCs. However, in cells co-transfected with Cx26S17F and Cx30, the fluorescence recovery rate was almost absent, suggesting that Cx26S17F exerted a *trans-*dominant-negative effect over Cx30 (*p* < 0.0001), forming non-functional GJCs (Figure 4B; Supplementary Table S9).

### 3.5 Hemichannels formed in cells co-expressing Cx26S17F and Cx30 are hyperactive and less sensitive to extracellular Ca^2+^ and La^3+^

**FIGURE 5:**
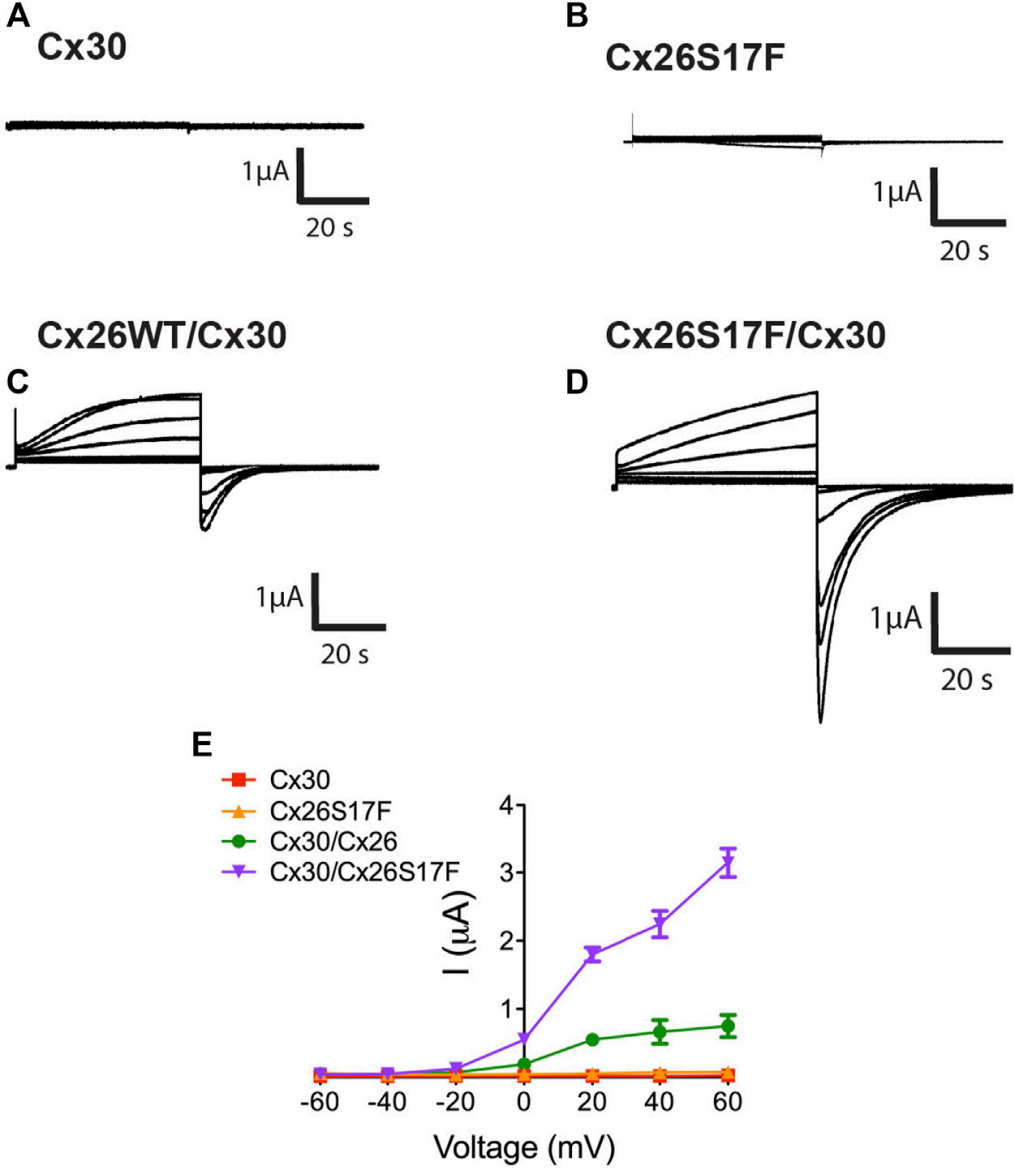
Heteromeric hemichannels formed by Cx26S17F/Cx30 induce strong currents in Xenopus oocytes. **(A,B)**. HC currents in oocytes co-expressing Cx30 **(A)** or Cx26S17F **(B)**. Representative membrane currents in response to depolarizing voltage steps from a holding potential of -10 mV and stepped in 20 mV increments from −60 mV to +60 mV. **(C, D)**. HCs currents in oocytes co-expressing Cx26WT/Cx30 **(C)** or Cx26S17F and Cx30 **(D)**. **(E)**. Graph representing the current/voltage relationship obtained from HC macroscopic currents. Data points represent mean ± SEM. *n* = 3.

The functional state of HCs formed by Cx26S17F and Cx30 was studied using different experimental approaches. We measured the macroscopic currents in *Xenopus* oocytes expressing Cx30 (Figure 5A), Cx26S17F (Figure 5B), or co-expressing Cx26 and Cx30 (Figure 5C) or Cx26S17F and Cx30 (Figure 5D) and bathed in normal extracellular solution containing 1.8 mM Ca^2+^. Oocytes expressing Cx30 (Figure 5A) showed negligible currents when depolarizing voltages steps were applied (Figure 5E), like the absence of HCs activity recorded in oocytes expressing the Cx26S17F mutation (Figure 5B) and consistent with a previous report (García et al., 2015). By contrast, oocytes expressing Cx26 showed a slowly activating current upon depolarizing step voltages, exhibiting a peak current at 30 mV and a subsequent current decay due to the activation of the V_j_ gating (García et al., 2018b). Interestingly, oocytes co-expressing Cx30 and Cx26S17F exhibited large HCs currents that were two-three-fold larger than those recorded in oocytes co-expressing the WT variants (Figures 5C, D), suggesting the formation of hyperactive heteromeric HCs (Figure 5E). In addition, current saturation at positive voltages pulses over 40 mV was not observed in heteromeric Cx26S17F/Cx30 HCs (Figure 5D), as is clearly observable in WT heteromeric HCs (Figure 5C), suggesting impairment in the closing gating mechanism associated with hyperactivity in mutant heteromeric HCs.

**FIGURE 6:**
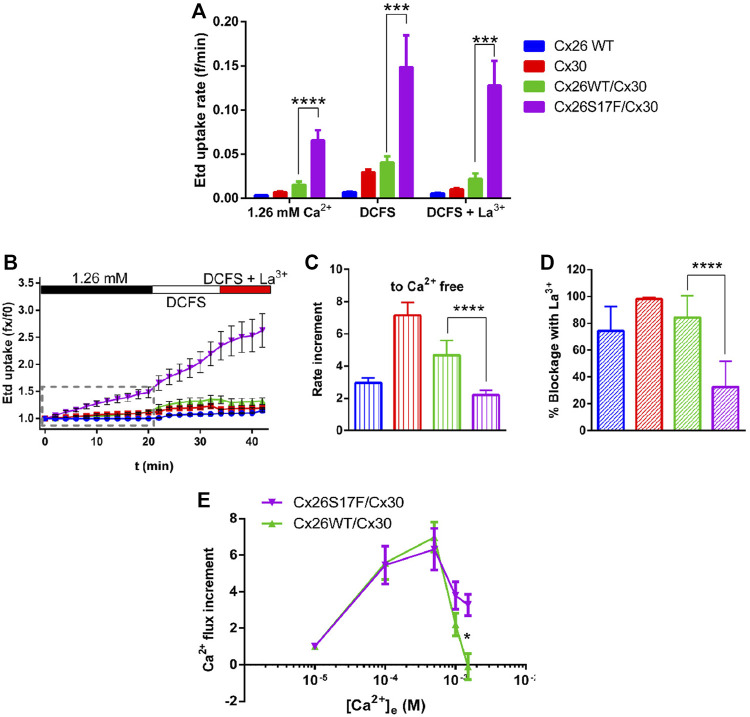
Heteromeric hemichannels formed by Cx26S17F/Cx30 have a hyperactive function and are insensitive to the Cx HC blockers La^3+^ and Ca^2+^. **(A)**. Consolidated data of dye uptake experiments. HeLa cells transfected with indicated Cx-constructs were bathed in Hanks solution containing 1 µM Etd, and 1.26 mM external Ca^2+^, 0 mM (nominal divalent cationic fee solution, DCFS), or DCFS plus 100 µM LaCl_3_ (DCFS + La^3+^). **(B)**. Representative Etd uptake in HeLa cells in basal, divalent cation-free solution (DCFS), and in DCFS +100 µM La^3+^. **(C)**. Relative changes in dye-uptake rate when [Ca^2+^]_e_ is reduced. **(D)**. Relative reduction of dye uptake after application of the HC blocker La^3+^. **(E)**. Normalized Ca^2+^ influx increment with respect to Ca^2+^ uptake rate at 10 μM. Statistical analysis, Mann-Whitney test **(A)**, *p < 0.05; **p < 0.005, ***p < 0.001; ****p < 0.0001 **(A–E)**. n = 13.

Since the uptake of Etd has mainly been used as an indicator of HCs activity (Johnson et al., 2016), we performed Etd uptake assays in HeLa transfectants bathed in a saline solution containing 1.26 mM Ca^2+^, in the absence of extracellular divalent cations (DCFS), or DCFS containing 100 µM of the non-selective Cx HC blocker, La^3+^ (Figures 6A–D). Cells expressing homomeric Cx26 or Cx30, as well as cell co-expressing Cx26 and Cx30 channels, showed very low Etd uptake rates in extracellular solution containing Ca^2+^ (Figures 6A, B). The cells co-expressing Cx26S17F and Cx30 showed large Etd uptake rates, which were almost four-fold larger than that of cells expressing HCs formed by Cx26 and/or Cx30 (Figures 6A, B). Moreover, in DCFS, most cells expressing Cx26, Cx30, or Cx26/Cx30 showed increased Etd uptake. The Etd uptake observed under both conditions described above was abolished by La^3+^ (Figure 6C). The high Etd uptake rate of cells expressing Cx26S17F/Cx30 showed only a moderate increase in DCFS, and this dye uptake was not significantly affected by La^3+^ (Figures 6A–D). These results strongly support the hypothesis that cells co-expressing Cx26S17F and Cx30 form hyperactive heteromeric HCs with altered gating and regulation properties.

### 3.6 Co-expression of Cx26S17F and Cx30 induces Ca^2+^ overload and cell damage

Since Cx26WT HCs are permeable to Ca^2+^ (Fiori et al., 2012), and high extracellular Ca^2+^ concentrations reduce the activity of Cx HCs (Sánchez et al., 2010), we evaluated the permeability of the plasma membrane to Ca^2+^ and its sensitivity to extracellular Ca^2+^ concentration. Intracellular Ca^2+^ signal rate (calcium influx) was measured in Fura-2 loaded HeLa cells co-expressing Cx26 and Cx30 or Cx26S17F and Cx30 under increasing [Ca^2+^]_e_ starting from 10 µM (Figure 6E). Both Cx combinations showed similar Ca^2+^ influx increments at 100 and 500 µM [Ca^2+^]_e_. However, at one and 1.5 mM [Ca^2+^]_e,_ the influx of Ca^2+^ in cells co-expressing Cx26WT and Cx30 importantly declined and reach a nadir at 1.5 mM [Ca^2+^]_e_, while in cells co-expressing Cx26S17F and Cx30, the Ca^2+^ influx persisted at higher [Ca^2+^]_e_ (Figure 6E). These results suggest that putative heteromeric HCs formed by Cx26S17F/Cx30 present a lower sensitivity to extracellular Ca^2+,^ and thus, they could lead to Ca^2+^ overload.

**FIGURE 7:**
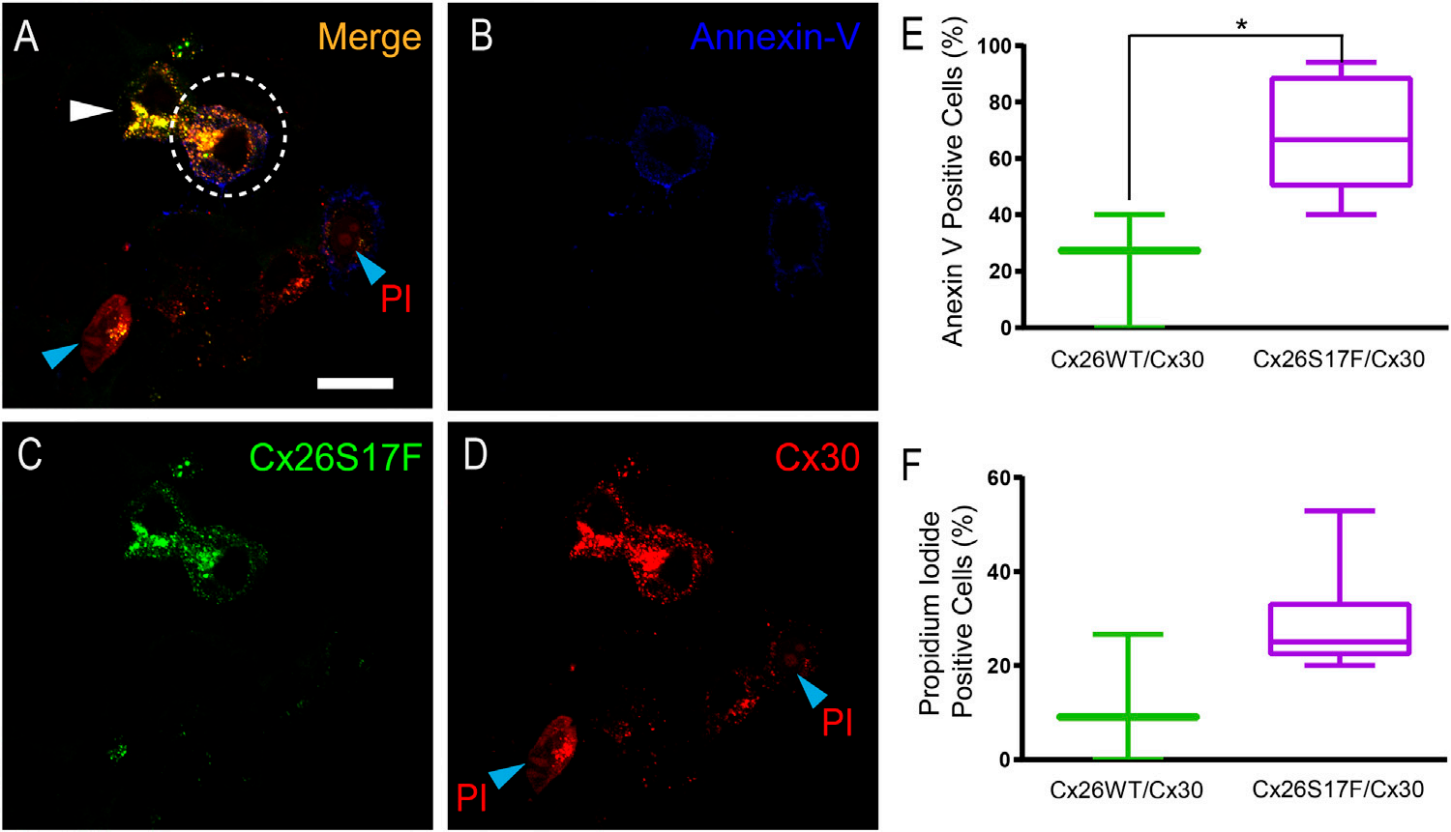
Expression of Cx26S17F/Cx30 increases cell damage and cell death in HeLa cells. **(A–D)**. Positive signal of annexin V at the membrane (blue, and **(B)** and PI [nuclear red stain, and **(D)**] at the nucleus of cells co-transfected with Cx26S17F (green, and **(C)** and Cx30-mCherry [cytoplasmic and membrane red signal, and **(D)**]. Dotted ellipse demarks a co-transfected cell with annexin V at the membrane, while the cell demarked with the white arrowhead is also co-expressing the two Cxs but it is in good shape. The cell at the bottom, demarked with a cyan arrowhead shows PI positive signal and co-expression of both Cxs, and rounded morphology as observed in dead cells. **(E)**. Percentage of transfected HeLa cells positive for Annexin V signal, indicative of cell damage and death. Statistical analysis, *p < 0.05. **(F)**. Percentage of transfected HeLa cells positive for PI, indicative of necrosis. Supplementary Figure 9 shows expression of annexin V and PI in cells expressing Cx26WT/Cx30.

As increments in [Ca^2+^]_i_ are associated with cell damage and death, we used the annexin V/propidium iodide (PI) assay to determine whether the Ca^2+^ imbalance observed in cells co-expressing Cx26S17F and Cx30 caused apoptosis or necrosis (Figure 7; Supplementary Figure S9). The assay was performed 16 h post-transfection, and ∼20% of HeLa cells co-expressing Cx26 and Cx30 were labeled with annexin V, and ∼11% were positive for PI, values that were like those observed in non-transfected HeLa cell cultures. In contrast, in HeLa cells co-expressing Cx26S17F and Cx30, more than 65% of the cells were positive for annexin V (Figures 7A–E), and ∼30% were positive for PI, indicating that some damaged cells developed necrosis (Figure 7F).

### 3.7 Molecular dynamics simulation shows alteration in Ca^2+^ coordination residues at the extracellular side of a putative heteromeric HCs formed by Cx26S17F/Cx30

**FIGURE 8:**
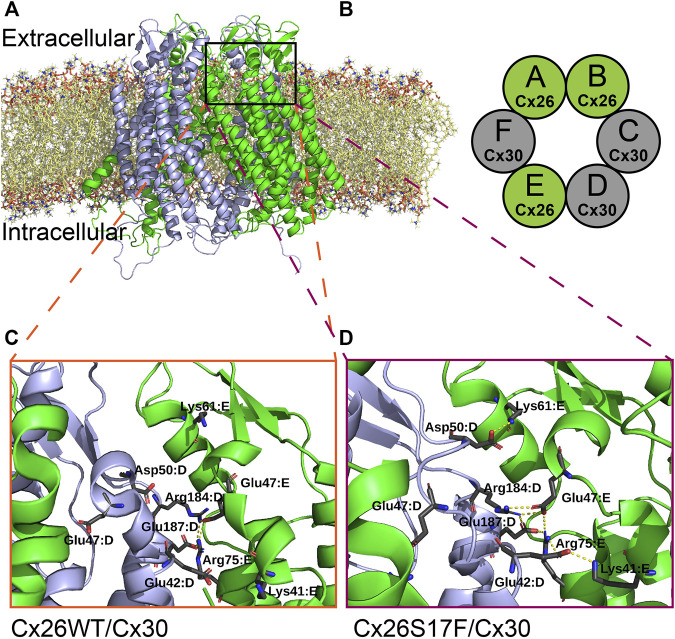
Possible new interactions in the mutant heteromeric HCs alters regulation by extracellular Ca^2+^. **(A,B)**. Molecular dynamic simulation of a heteromeric HC composed by Cx26 or Cx26S17F and Cx30 **(A)**, according to the most frequent combination (stoichiometry) of each monomer in the heteromeric hemichannel **(B)**, as described in Naulin et al., 2020, which is Cx26-Cx26-Cx30-Cx30-Cx26-Cx30. Colors indicate Cx26 or Cx26S17F in green and Cx30 in grey. **(C,D)**. Zoom of the interaction network around parahelix highlighted in **(A)**. The residues forming salt bridges are shown in sticks with the corresponding label. In the top of the figure the interaction between Asp50 of chain D (Cx30) with Lys61 of the next chain (Cx26), which has been proposed to be associated with the stabilization of open state, appears in Cx26S17F/Cx30 HCs **(D)**, and does not occur in Cx26WT/Cx30 HCs **(C)**.

To get some molecular insights into the mechanism of action of the hyperactive HCs, molecular dynamics simulations were run for putative heteromeric HCs formed by Cx26S17F/Cx30 (Figure 8). Heteromeric HCs were built for Cx26S17F/Cx30 and Cx26WT/Cx30 using the most probable stoichiometry found by Naulin et al. (2020), which the hexamer is formed by the following subunit combination: Cx26:Cx26:Cx30:Cx30:Cx26:Cx30. Both HCs, with Cx26WT and Cx26S17F, showed similar behavior, maintaining their overall structure and inner diameter.

We studied the polar interaction network inside each structure during the simulation to explore a possible mechanistic explanation for the loss of calcium regulation in S17F (Figures 8A, B). We detected the forming of some inter-monomer salt bridges involving several residues in the last three monomers (Figure 8D), which include an alternating pattern for heteromeric Cx26S17F/Cx30 HCs (Figure 8B). These salt bridges are not present in the Cx26WT version of the HCs (Figure 8C). One of the new interactions is an interaction between D50 from monomer D with K61 from monomer E (Figures 8C, D Cx30 (grey) and Cx26S17F (green), respectively). This interaction was proposed as a stabilizer of the open state (Lopez et al., 2013, 2016). On the other hand, residue E42 from monomers D and E established salt bridges with residues K41 and R75 of monomers E (Cx26S17F, green) and F (Cx30, not shown), respectively. These interactions were not detected in WT HCs but are part of a bigger interaction network already described in this region of homomeric connexin channels (Kwon et al., 2012; Lopez et al., 2016; García et al., 2018a). E42 is also one of the coordinators of calcium (Bennett et al., 2016); changes in its interaction network could be related to a change in the affinity for Ca^2+^ or other HC blockers, consistent with affectation in the HCs gating mechanism.

## 4 Discussion

In the present work, we found that the expression of Cx26S17F in cochlear explants caused the formation of hyperactive HCs and less-functional GJCs in supporting cells of the organ of Corti, was associated with damage in sensory hair cells stereocilia, and would explain the deafness phenotype. Further study will be required to demonstrate this possibility. The hyperactive HCs are insensitive to blockage by classical HCs blockers, like extracellular Ca^2+^ and CBX. We observed the formation of hyperactive HCs and non-functional GJCs in cells co-expressing Cx26S17F and Cx30, which, together with molecular (PLA) and co-localization analysis, suggest the formation of heteromeric channels between Cx26S17F and Cx30 in HeLa cells. These putative disease-associated heteromeric HCs may present reduced sensitivity to extracellular Ca^2+^, which prevents closure of the HCs and allows a significant influx of Ca^2+^ into cells resulting in some cellular damage, but not necessarily cell death. Molecular dynamic experiments in hypothetic heteromeric HCs formed by Cx26S17F and Cx30 suggest that altering extracellular Ca^2+^ binding sites and gating mechanisms can produce HCs hyperactivity.

To study the consequences of KID mutations *in-vivo,* two transgenic mouse lines have been developed (Schütz et al., 2011) (Mese et al., 2011), and these studies have focused mainly on the skin phenotype (García et al., 2016a). Here, we used the same Cx26S17F mouse line as Schütz et al. (2011), but it was crossbred with CMV-Cre mice to induce a KID phenotype (KI Cx26S17F) in cochlea and skin. Moreover, the expression of the Cx26S17F variant was accomplished by *in vitro* treatment with recombinant TAT-Cre of cochlea explants from the Cx26S17F mouse line (cKI Cx26S17F). These models revealed that expression of a deafness syndromic mutant, Cx26S17F, causes the formation of hyperactive HCs and less functional GJCs in supporting cells of the organ of Corti. In fact, *in situ* expression of Cx26S17F in supporting cells in explants of the organ of Corti (cKI Cx26S17F) was enough to induce degeneration of hair cell stereocilia, which indicates that supporting cells keep a dynamic control of hair cell function.

It has been proposed that Cx HCs play important roles in cochlear development (Wingard and Zhao, 2015), and in physiological or pathological cochlear function (Verselis, 2017). Thus, the expression of hyperactive HCs could severely impact the cell viability and function of cochlear supporting and skin cells since HCs, in part, mediate ATP release from these cells and modulate paracrine signaling between support and hair cells in the cochleae (Zhao et al., 2005) and activate an inward K^+^ current into supporting cells, which has been suggested to be important for the excitability of hair cells (Zhu and Zhao, 2010). A similar mechanism has been proposed for skin cells (Skinner et al., 1981; Sanchez and Verselis, 2014).

Because the expression of Cx26S17F in exogenous expression systems did not develop functional GJCs or HCs (García et al., 2015), it is possible that interaction of Cx26S17F with other WT Cxs co-expressed with mutant Cx26 in the cochlea, like Cx30, cause the formation of functional aberrant channels. Indeed, we observed a strong co-localization of Cx26S17F and Cx30 in several cochlear-supporting cells. Moreover, we can not discard that the differences in the magnitude of the effects induced by the expression of Cx26S17F on the DAPI uptake between different supporting cell types can be the consequence of differences in the expression ratio between Cx26 and Cx30, in fact Deiters cells express more Cx30 than Cx26 (Figure 1), and this is consistent with previous publications (Sun et al., 2005; Ortolano et al., 2008).

The finding that Cx26S17F affects the trafficking of Cx30 is consistent with other KID mutations in Cx26, such as G12R and D50N, that also present trafficking defects (Common et al., 2003; Meşe et al., 2007; García et al., 2015). Similar results were obtained in Cx31 mutations that cause other skin disorders (Meşe et al., 2007). These findings support a common mechanism of syndromic deafness mutations with skin phenotype that involves the mislocation of hyperactive or aberrant HCs. Recently, Defourny and Thiry (Defourny and Thiry, 2021) proposed that cadherin-based tricellular adherens junctions, a specialized cell-cell junctions formed at sites where three epithelial cells make contact, which is enriched in lipid rafts, promote the microtubule-mediated trafficking of Cx26/Cx30 heteromeric channels to the cell surface and further assembly into GJ plaques in cochlear supporting cells. We cannot discard that mutant heteromeric Cx26S17F/Cx30 channels miss target tricellular junctions causing less GJ plaque formation. Further studies will be necessary to clarify this point.

Although we did not directly demonstrate the formation of heteromeric channels between Cx26S17F and Cx30, the most plausible interpretation of data obtained by co-localization, PLA, functional assay (for HCs and GJCs), all support the formation of heteromeric channels between Cx26S17F and Cx30. This is consistent with previous studies that demonstrate the formation of heteromeric channels between Cx30 and Cx26 using co-immunoprecipitation, co-localization and functional studies (Sun et al., 2005; Yum et al., 2007; Ortolano et al., 2008). Our results indicate that even though S17F mutation changes the ability of Cx26 to interact with Cx43 (García et al., 2015), it does not significantly affect the interaction of mutant Cx26 with Cx30, supporting the notion that there are other Cx compatibility signals beside the N-terminal domain (Jara et al., 2012; García et al., 2015). In addition, the expression of Cx26S17F produces a *trans*-dominant-negative effect over GJCs formed by other Cxs, like Cx43 (García et al., 2015) or Cx30 as shown here. Confirming that a common feature in non-syndromic and syndromic deafness is that mutant forms of Cx can affect the function of co-expressed WT Cxs and result in loss of intercellular coupling or the formation of GJCs with altered permeability to second messengers such as IP_3_, while maintaining the normal transfer of ionic current through GJCs (Gossman and Zhao, 2008; Yum et al., 2010; García et al., 2016b). Although reduced GJC activity can be critic for the proper function of the cochlea, the exact role of GJCs in cochlear function and development is yet unknown. The cochlear sensory epithelium is a poorly vascularized tissue (Pickles, 2015). Looking at a sagittal section of the *scala media*, the irrigated part of the tissue would only correspond to the *stria vascularis*, where the perilymph is generated from blood serum (Wan et al., 2013). Therefore, it has been suggested that the GJC network connecting supporting cells and the network in the lateral wall is the principal way for cells to obtain nutrients and molecules relevant to the correct functioning of the auditory tissue (Zhao et al., 2005; Johnson et al., 2017; Chen et al., 2018; Ceriani et al., 2019). As speculation, if the GJC network is not working properly, recycling of K^+^, intercellular IP_3_, ATP, and Ca^2+^ diffusion, among other signaling molecules or metabolites, would be altered, affecting the proper functioning of this tissue (Piazza et al., 2007; Majumder et al., 2010). Consistently, some animal models of non-syndromic deafness present a lack or reduction in GJC function (Johnson et al., 2017).

Although the recording conditions used in this study to determine the functional state of HCs and GJCs are identical to most studies in the field, they are non-physiological; hence we cannot discard that some physiological parameters, like Cx26 sensitivity to CO_2_, could potentially affect the results. For example, some Cx26 KID mutants, like N14K, lost their regulation by CO_2_ (de Wolf et al., 2016). Cx26 has been demonstrated to be sensitive to CO_2_ and an increase in CO_2_ open HCs and closed GJ channels (Meigh et al., 2013; Nijjar et al., 2020) similar to our finding in cells expressing Cx26S17F/Cx30. The action of CO_2_ on Cx26 has been proposed to occur *via* carbamylation of the residue K125 and also requires R104 (Meigh et al., 2013). Although, K125 is located in the intracellular loop of Cx26, it seems that CO_2_ induces conformational changes that are transmitted to the N terminus to unfold it and plug the channel (Brotherton et al., 2022). Interestingly, KID mutations located in the NT of Cx26, like G12R and N14K, also produce aberrant interaction with residues in the intracellular loop (Sanchez et al., 2016; Garcia et al., 2018a). A similar mechanism could be proposed for Cx26S17F interacting with Cx30, probably by the formation of aberrant heteromeric channels that can alter the mechanism of gating conformation at the intracellular sites of connexons.

It has been proposed that some KID syndrome mutations may activate a pre-existing cryptic splice site in Cx26 mRNA in conjunction with a fluorescent protein tag (i.e. GFP) for chimeric construction (Cook et al., 2019) that may have toxic effects. However, previous study with this mutant using Cx26S17F cDNA constructs with or without GFP tag show the expression of full length Cx26S17F by sucrose gradients resolution and western blot analysis (Garcia et al., 2015). In addition, the data obtained in cochlear explants and HeLa cells are consistent with the increase in whole-cell HC currents observed in oocytes after co-injection of Cx26S17F and Cx30 mRNAs, suggesting that the gain-of-HC activity observed was not a consequence of the expression of splice variants of Cx26.

The co-expression of Cx26S17F with Cx30 produce HCs that did not respond properly either to extracellular Ca^2+^ or La^3+^, suggesting an abnormal gating by extracellular cations, which agrees with the functional properties of other KID mutations (García et al., 2016b). It has been proposed that Ca^2+^ ions are coordinated with residues D50 and K61 such that when Ca^2+^ is bound to these residues, it destabilizes the open state of HCs, favoring a closed conformation (Lopez et al., 2016). In addition, we and others have shown that the mutation G12R, N14K, and N14Y, which are also located at the NT, alters the fast and slow voltage gating, renders HCs with increased open probability, and poor regulation by [Ca^2+^]_e_ (Sanchez et al., 2016; García et al., 2018b). Although we cannot be certain that the same mechanism applies to the S17F mutation, both mutations have several functional hallmarks in common. The fact that the hyperactive HCs formed by Cx26S17F/Cx30 cannot respond to HC blockers such as La^3+^ or CBX suggests that the general closing mechanism of this channel is severely affected.

As an initial attempt to understand the loss of Ca^2+^ regulation by these putative heteromeric Cx26S17F/Cx30 HCs, we performed molecular dynamics simulation in WT and mutant heteromeric HCs. We found that Cx26S17F/Cx30 HCs present changes in inter-monomer salt bridges between extracellular-side residues of HCs, including D50, K61, E42, and R75, that are relevant for extracellular Ca^2+^ coordination that keeps HCs closed (Bennett et al., 2016; Lopez et al., 2016; García et al., 2018b). Thus, these molecular interaction network changes are consistent with the observed changes in extracellular Ca^2+^ sensitivity in Cx26S17F/Cx30 heteromeric HCs. However, molecular dynamic study was generated using as template the structure of Cx50/Cx46 cryo-EM from Flores et al. (2020) and not the last structure available of Cx6 (Brotherton et al., 2022), therefore, we cannot discard an alternative explanation to the hypothesis derived from our molecular dynamic analysis. Further studies using molecular dynamic simulation with new Cx26 structure available (Brotherton et al., 2022) and mutagenesis of the amino acid residues identified in Cx26S17F/Cx30 channels that may affect extracellular Ca^2+^ binding will be necessary to clarify this point.

Consistently, we found that cells expressing Cx26S17F/Cx30 HCs present higher [Ca^2+^]_i_ than cells expressing Cx26WT/Cx30 HCs, which is similar to previous findings in cells co-expressing Cx26S17F/Cx43 (García et al., 2015). This is relevant because persistent increases in [Ca^2+^]_i_ may lead to cell death, which can be part of the KID´s pathological features (Sánchez et al., 2010; Terrinoni et al., 2010; Decrock et al., 2011; Vinken et al., 2011). Moreover, increased [Ca^2+^]_i_ may be required for HC opening and GJC closure through interaction with Ca^2+^/calmodulin (CaM) that has been observed at around 500 nm [Ca^2+^]_i_ (de Vuyst et al., 2006; Peracchia, 2020). Although it is still unknown whether CaM binds Cx26 or Cx30, putative binding sites need to be explored.

This work strongly supports the hypothesis that the failure in “fine-tuning” of HCs caused by the Cx26S17F mutation undermined the cochlear and skin homeostasis and induced hearing loss and severe skin disease. Therefore, pharmacological targeting of hyperactive HCs can have therapeutic potential for skin disease and deafness conditions in KID syndrome.

## Data Availability

The raw data supporting the conclusions of this article will be made available by the authors, without undue reservation.
