# Supplementary material for: Expression of KID syndromic mutation Cx26S17F produces hyperactive hemichannels in supporting cells of the organ of Corti
Source: Front Cell Dev Biol. 2023 Jan 9;10:1071202. doi: 10.3389/fcell.2022.1071202 (PMC9868548; doi:10.3389/fcell.2022.1071202)
Supplement: Supplementary file 1 [file DataSheet1.pdf]

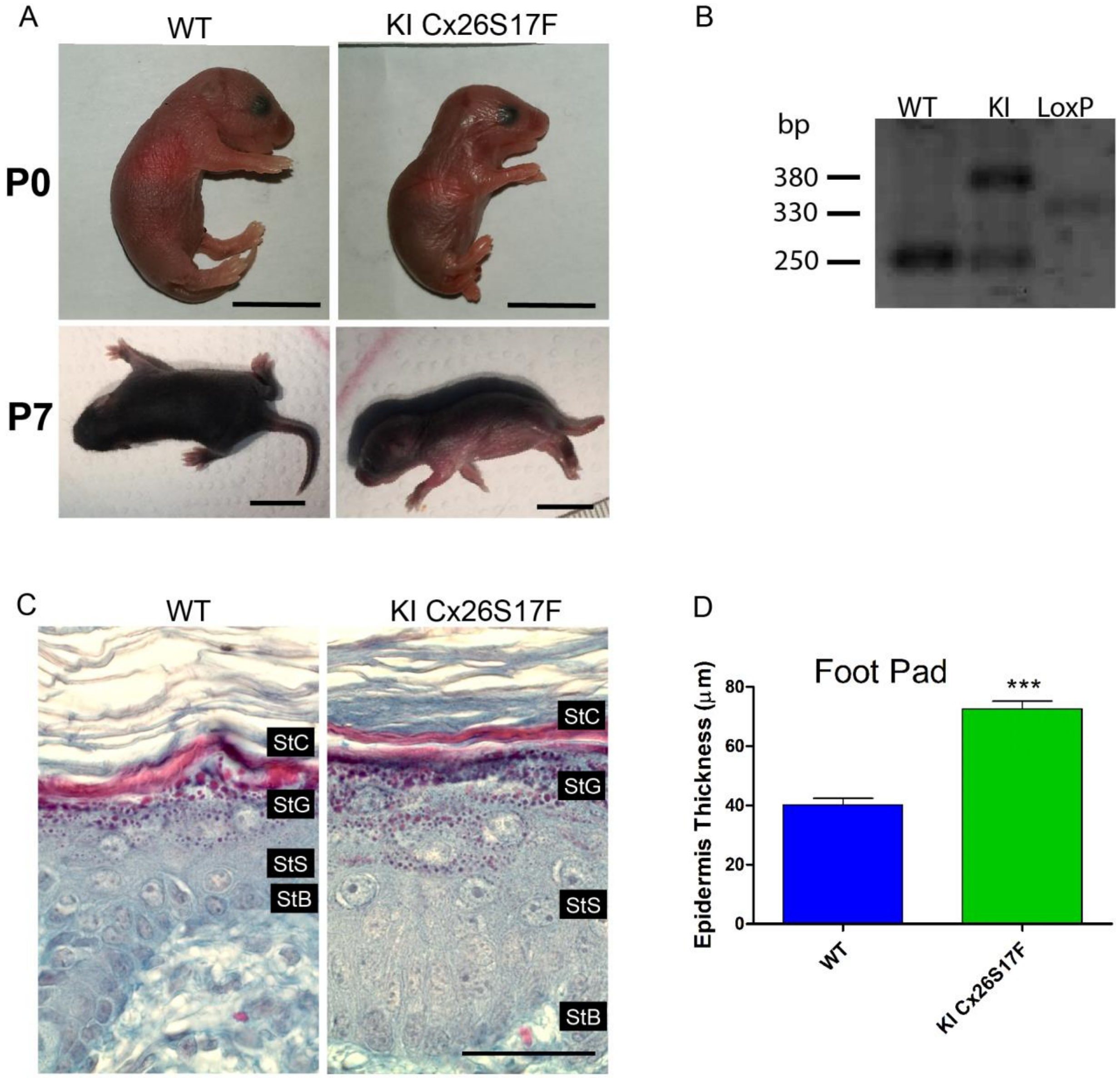

Supplementary figure S1. Transgenic KI Cx26S17F mice present aberrant skin phenotype.
A. Phenotype observed at P0 (upper panels) and at P7 (lower panels) in carrier mouse Cx26+/floxS17F (control) and KI Cx26S17F. **B.** DNA electrophoresis of WT, KI Cx26S17F (cKI) and Cx26+/floxS17F (LoxP) genotyping, as described in the Methods section, stained with etidium bromide. Using LoxP primers, WT allele is expected to show a 280 bp fragment, carrier Cx26+/floxS17F allele shows a 330 bp fragment and KI Cx26S17F allele a 380 bp fragment. **C.** Thicker epidermis present on the foot pad of transgenic mice (KI Cx26S17F), associated with hyperplasic spinosum and granulosum stratus of KID syndrome. **D.** Summary of analysis of epidermal thickness observed in the foot pad of WT (blue) and cKI Cx26S17F (green) mice. Scale bar 1cm (a) and 50µm (c). StC= stratum corneum; StG= stratum granulosum; StS= stratum spinosum; StB= stratum basale. Statistical analysis, paired t test ***$p < 0.001$.

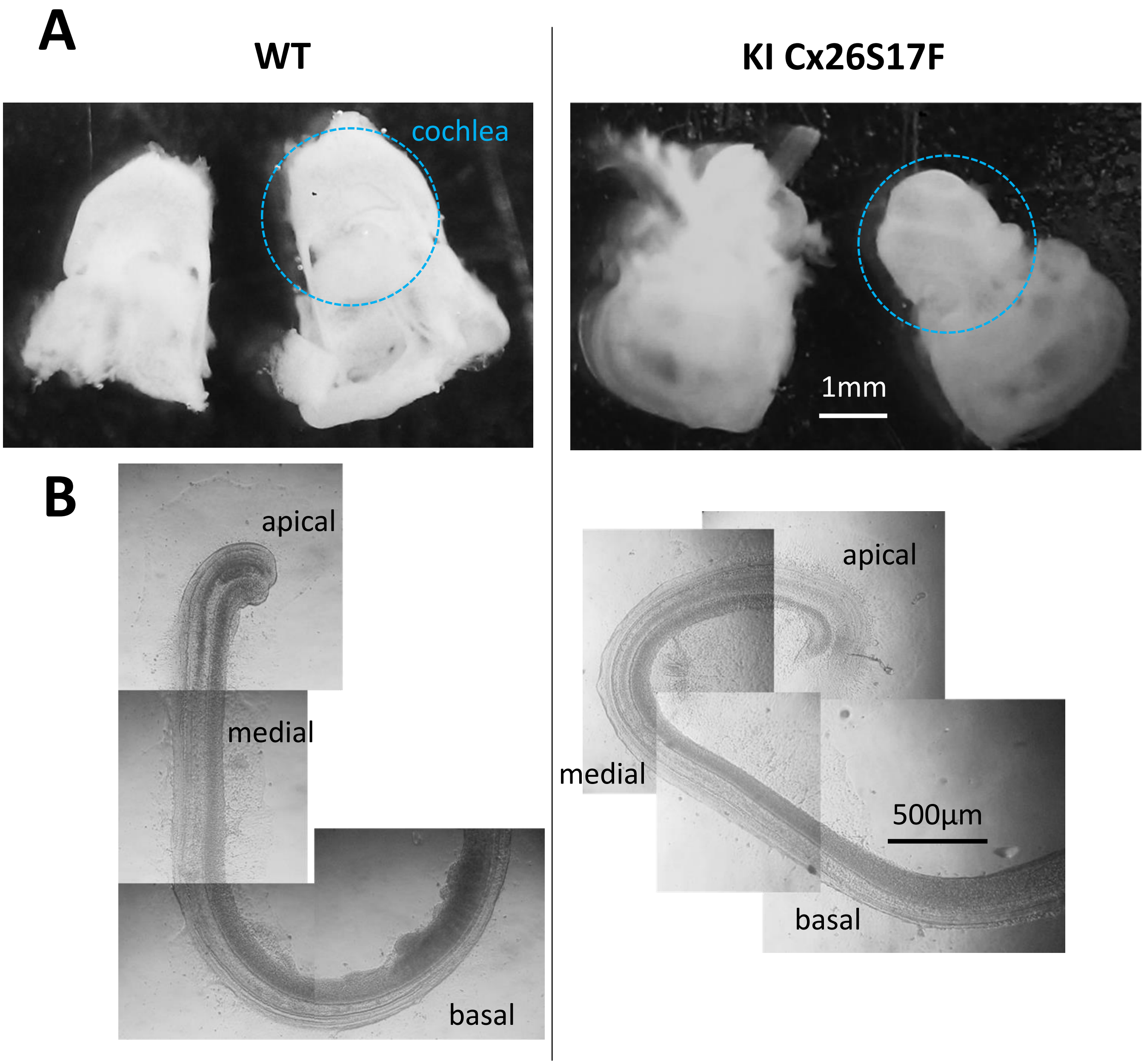

Supplementary Figure S2. KI Cx26S17F cochlear anatomy and explants show no major alterations. A) Cochleae extracted from animals obtained from the same litter, one with wild-type (WT) phenotype and the other with KI Cx26S17F phenotype. *Blue circle* circumscribes a cochlea from WT animal to show its larger size with respect to the cochlea from a KI Cx26S17F animal. No other changes in the cochlear or vestibular system were observed. C) Explants of the organ of Corti derived from the same cochleae shown in B. No major changes in the macroscopic structure of the explant or signs of cell damage or death are observed. After 2 days *in vitro*, no signs of detachment from the substrate were observed in both tissues.

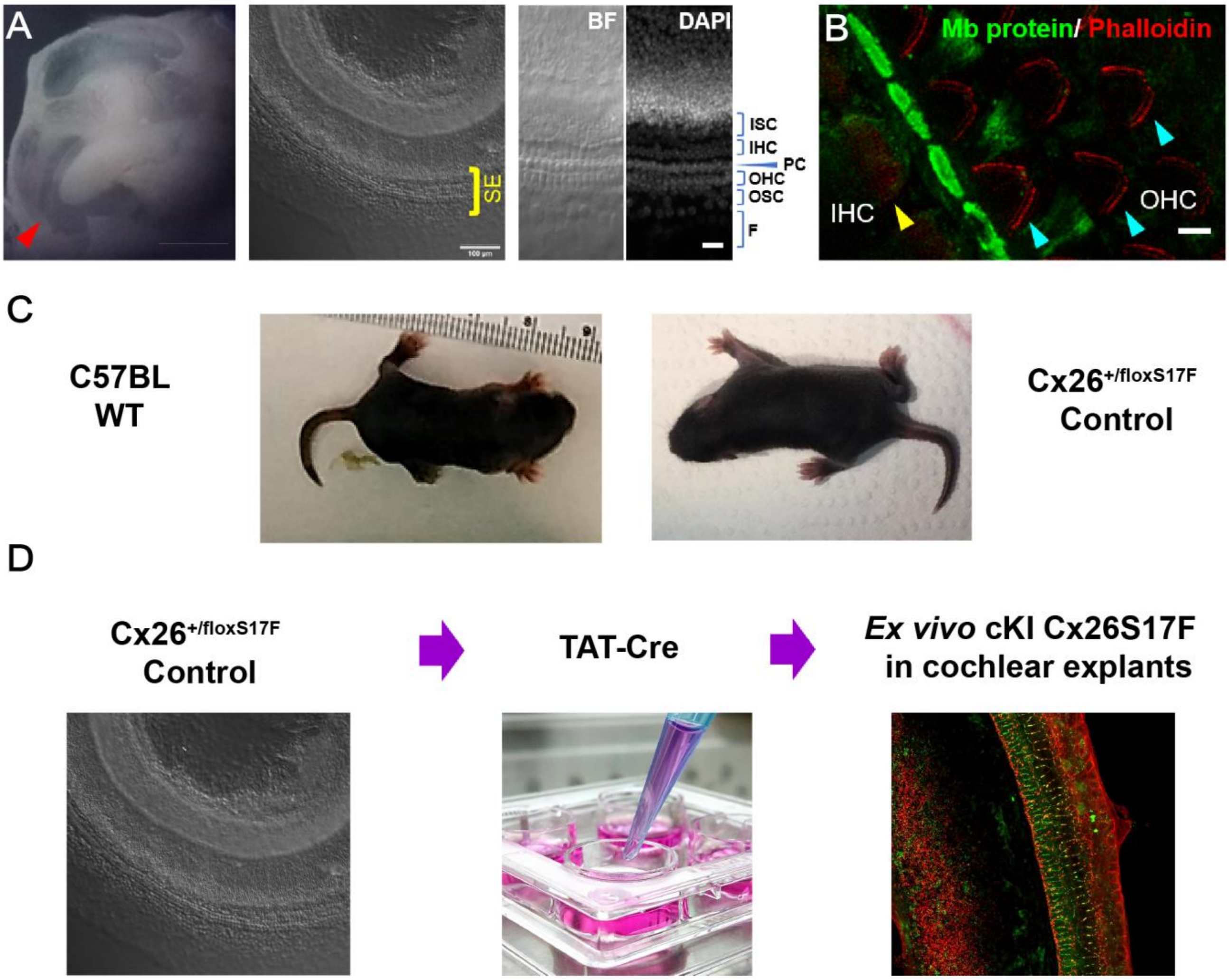

Supplementary Figure S3. Ex-vivo model for the study of Cx26S17F in the cochlea.
A. Preparation of organ of Corti explants. Extraction of cochlea and sensory epithelium (organ of Corti) followed by maintenance of the explants for up to 2 days in vitro. BF= Bright field, ISC= Inner supporting cells, IHC= Inner hair cells, PC= Pillar cells, OHC= Outer hair cells, OSC= Outer supporting cells, F= Fibrocytes. **B.** Phalloidin labeling of stereocilia in hair cells to show subcellular integrity of the preparation. IHC shown in another plane. Mb= membrane. **C.** Phenotype of WT (C57BL, left) and carrier mice (Cx26+/floxS17F) showing no difference at P7. **D.** *In vitro* induction of mutant Cx26S17F in cochlear explants of carrier mice using TAT-Cre. Scale bars: (a) left panel 0.5 mm; middle, 100 µm; right panel 20 µm. (b) 2 µm.

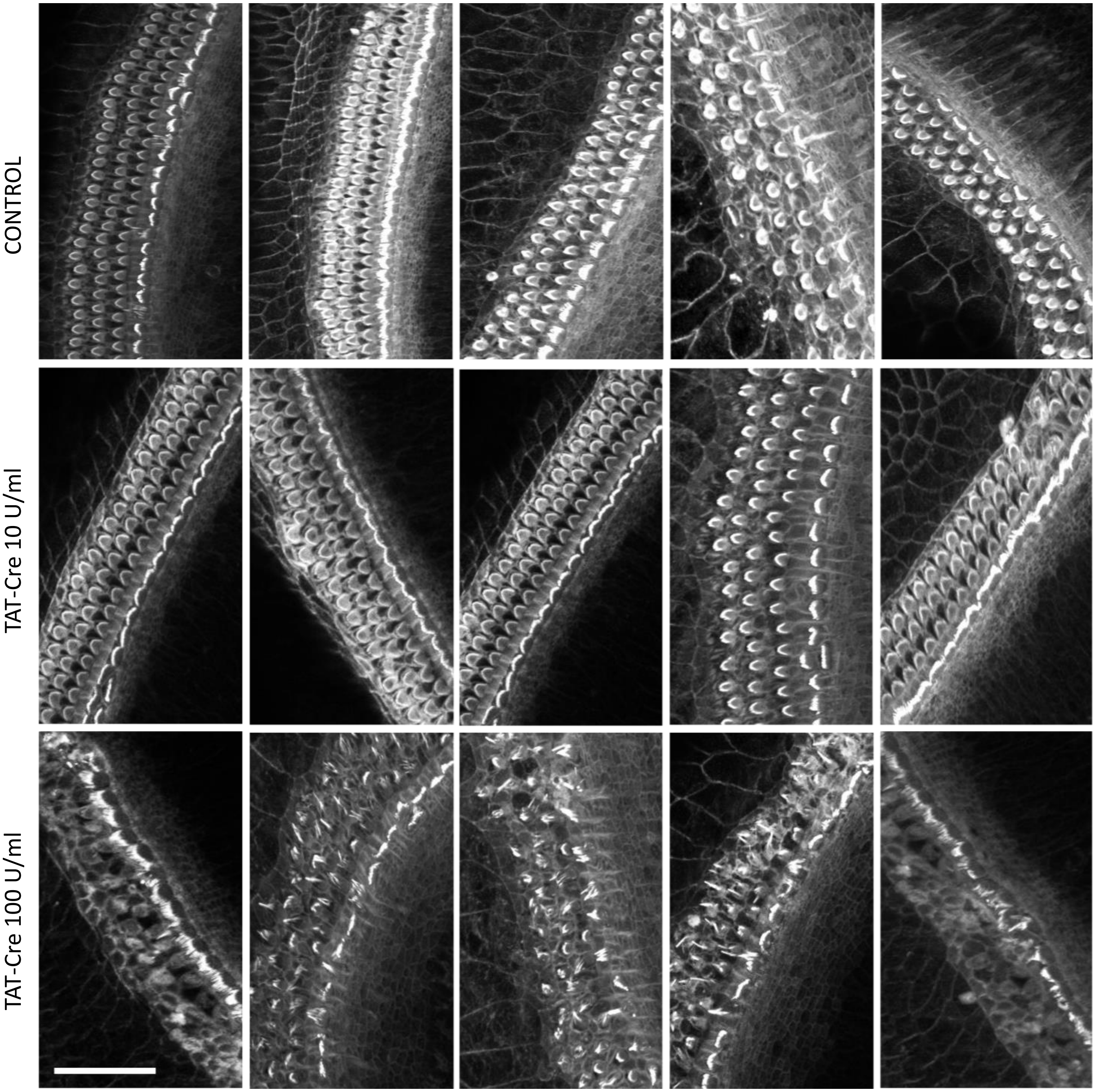

Supplementary Figure S4. Treatment with 100 U/ml, but not 10 U/ml, of TAT-Cre produce disorganization of OHC´s stereocilia. Cochlea cultures form WT animals were untreated (Control) or treated with 10 or 100 U/ml of TAT-Cre recombinant peptide for 24 hrs. Stereocilia were stained with phalloidin as indicated in methods. Each panel show a representative fluorescent confocal images (Z projections) of different cochleae at the level of hair cells rows for each condition. Only a higher dose of TAT-Cre (100 U/ml) produce disorganization of OHC´s stereocilia with none or minor effect over IHC´s stereocilia. Calibration bar = 50 µm

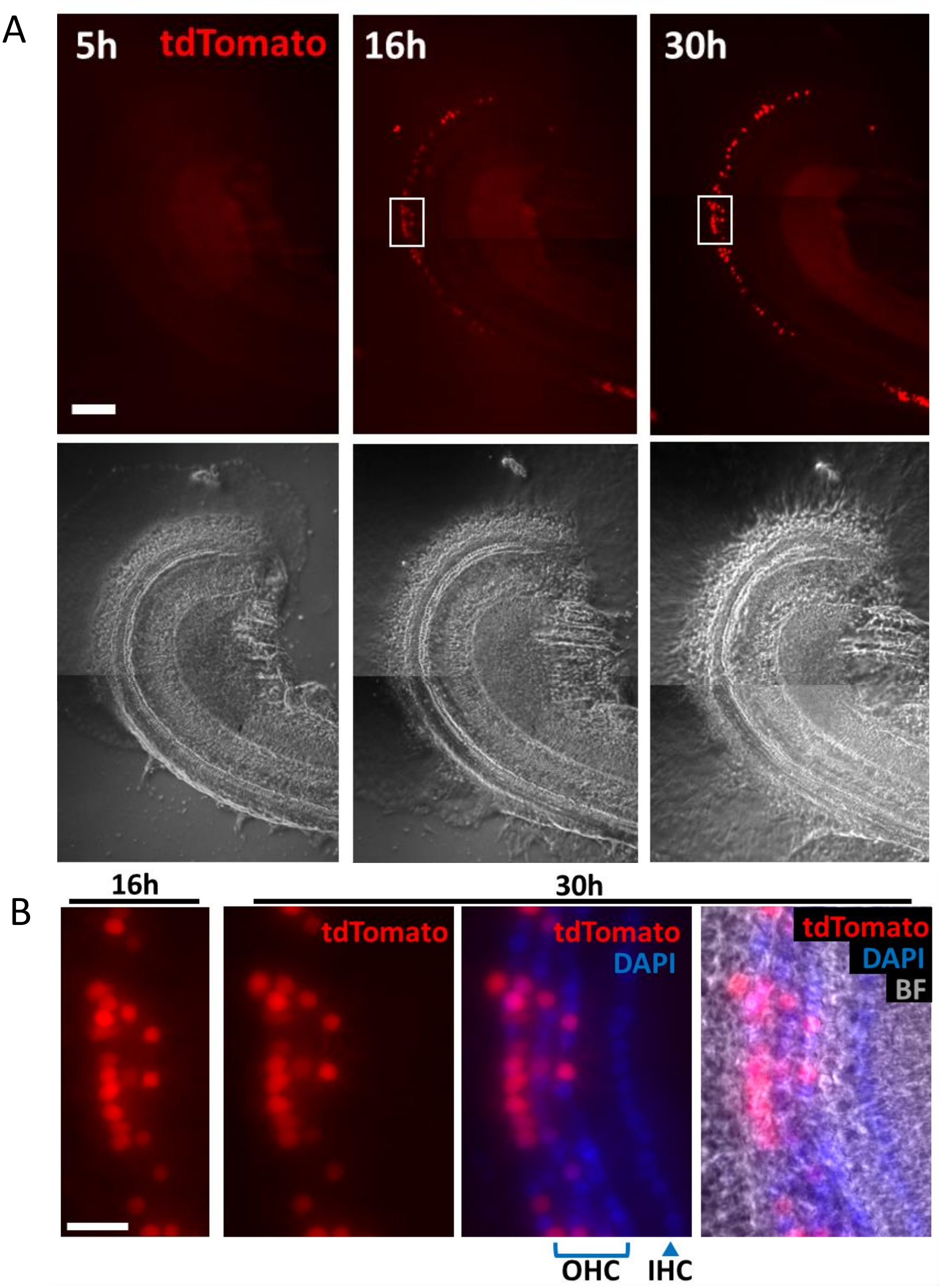

Supplementary Figure S5. TAT-Cre induced recombination in cochlear explants, using tdTomato as a reporter. **A.** *Upper panels*. tdTomato expression induced by application of recombinant TAT-Cre protein on cochlear explants obtained from P7 Ai9(RCL-tdT) mice (Jackson Laboratory, #007907; kindly donated by Dr. Chiayu Chiu). Expression of tdTomato is evident at 16h and 30h post-application of enzyme. *Lower panels*, bright field pictures, showing explant development. **B**. Details of the region indicated in (a), at 16h and 30h, respectively. At 30h, the explant was exposed to 1 µM DAPI, to indicate cell position. Bright field (BF) is shown for reference in the right panel.

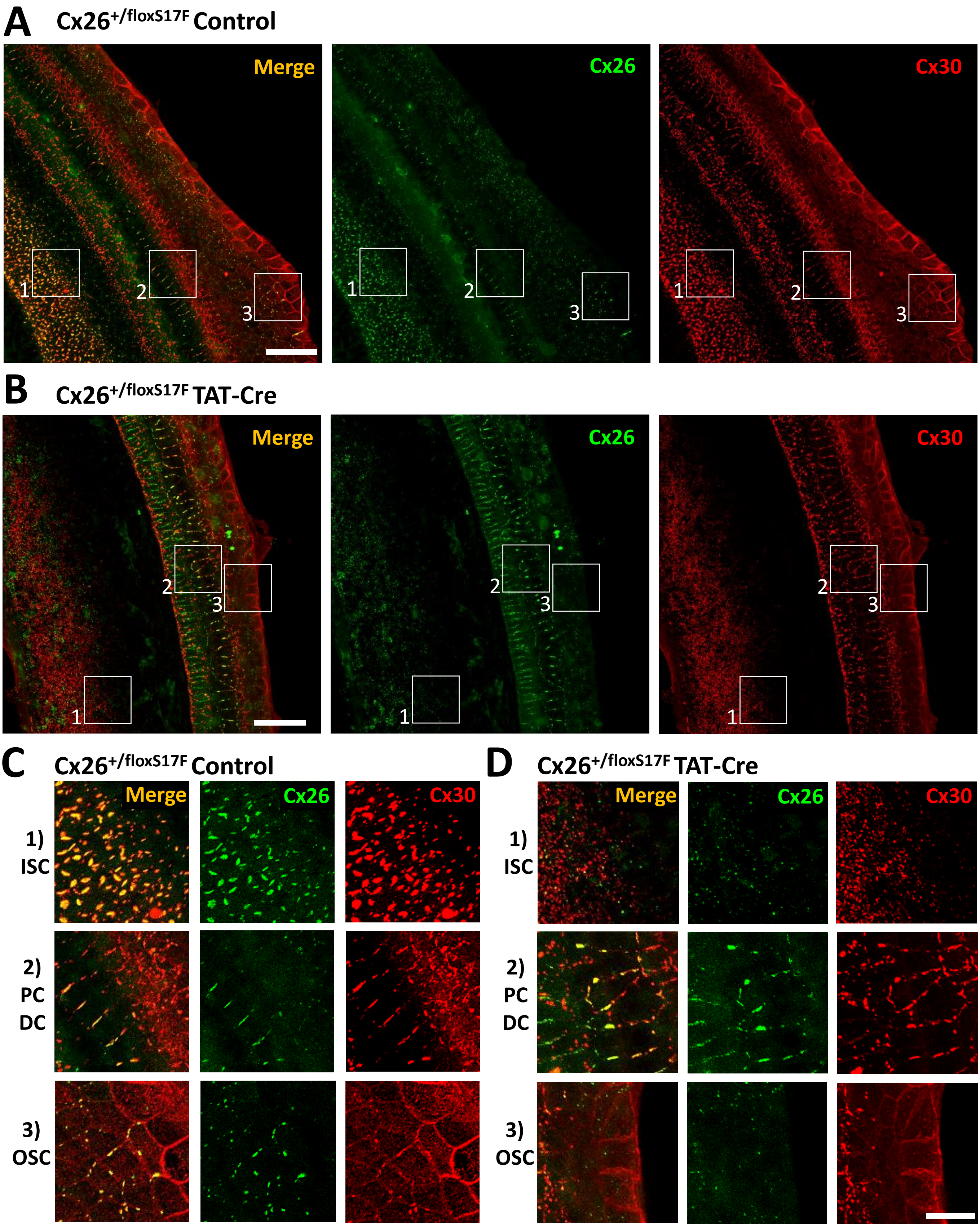
A
Cx26+/floxS17F Control
Merge
Cx26
Cx30
1
2
3
B
Cx26+/floxS17F TAT-Cre
C
Cx26+/floxS17F Control
1) ISC
2) PC DC
3) OSC
D
Cx26+/floxS17F TAT-Cre

Supplementary Figure S6. Expression of Cx26S17F in cochlear explants changed the distribution of Cx26 and Cx30 in supporting cells of the organ of Corti. A, C. Control cochlea ($Cx26^{+/floxS17F}$ Control) showed the typical distribution of Cx26 (green) and Cx30 (red), forming co-localizing GJ plaques, as seen in the magnified views of ISC, PC and DC and OSC (c). **B, D**. Expression of cKI Cx26S17F induced by the addition of TAT-Cre ($Cx26^{+/floxS17F}$ TAT-Cre) changed the distribution of mutant Cx26S17F and Cx30. Magnified views are shown in (d), where GJ plaques are reduced and both Cxs are retained in the intracellular space in ISC. In addition, OSC expression of GJ is totally lost. PC and DC did not show any differences. Scale bars: 50 µm (a,b), 15 µm (c,d). The different signals are displayed separately to highlight the loss of colocalization of Cx26-S17F and Cx30.

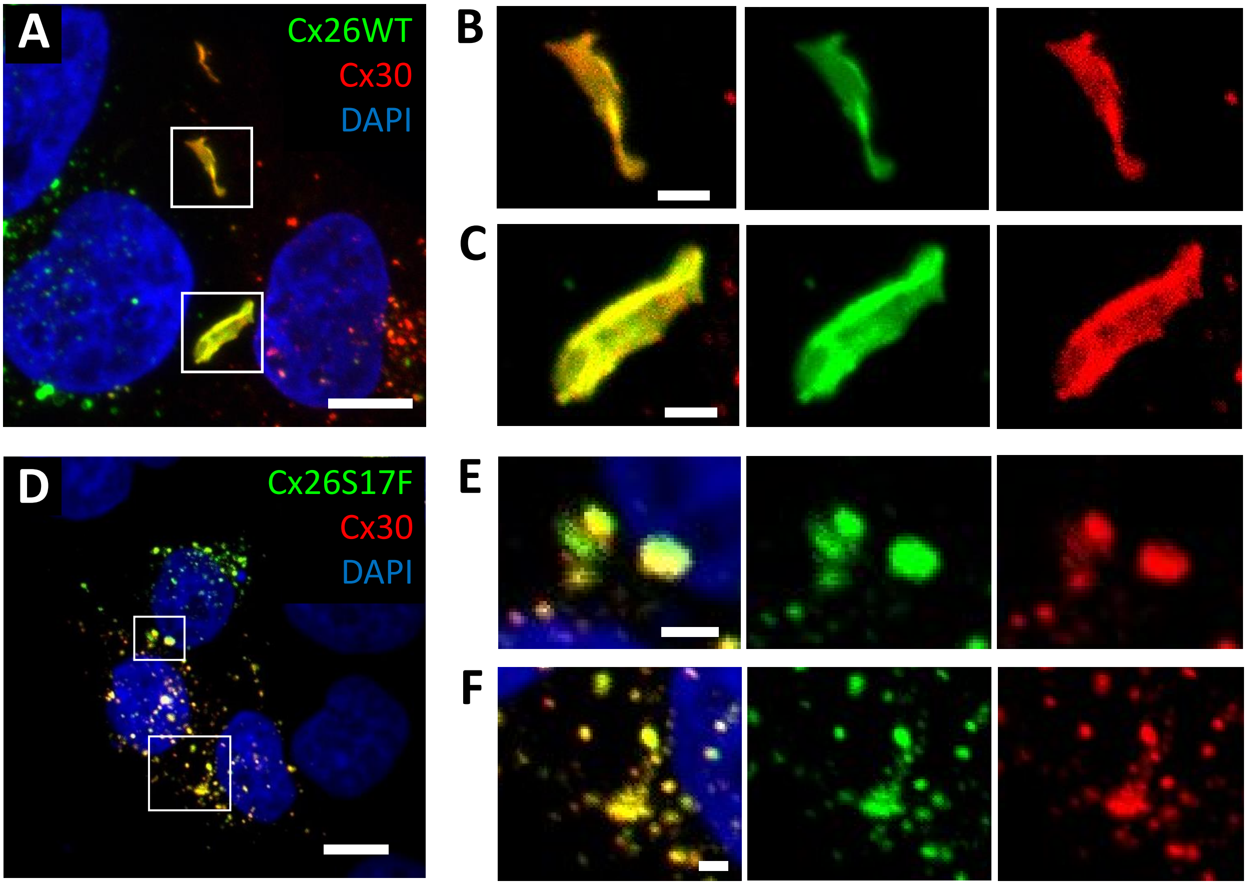

Supplementary Figure S7. Mutant Cx26S17F co-localizes with Cx30 and changes the distribution of heteromeric hemichannels and gap junctions in HeLa cells. A. Strong co-localization of Cx26WT (green) and Cx30 (red) in GJ plaques. **B-C.** Zoom of GJ plaques observed in A. **D.** Strong co-localization of Cx30 (red) with mutant Cx26S17F (green) was observed mainly in intracellular compartments, and occasionally in a few small plaques in the appositional zone. **E-F.** Zoom of small GJ plaques (E) and intracellular (F) co-localization between Cx30 and mutant Cx26S17F. Scale bar: 10 µm (A, D) and 2 µm (B, C, E and F).

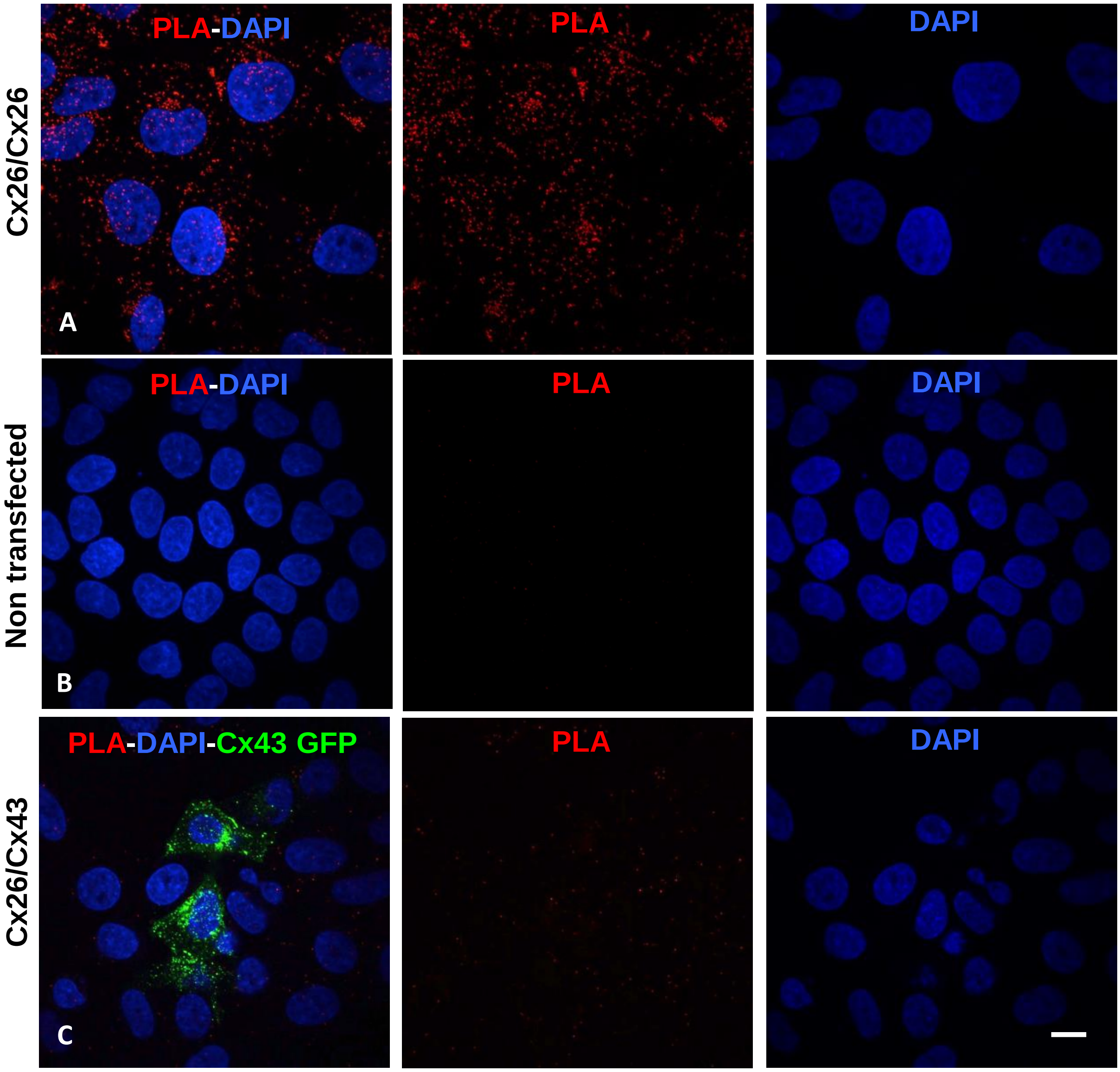

Supplementary Figure S8. Proximity ligation assay internal controls. Using the same dilutions for each antibody described in the method section, we performed positive and negative controls on transfected and non transfected HeLa cells as indicated below. A) A stable line of Cx26 HeLa cells was used as a positive control for PLA assay, using two different anti Cx26 antibodies (mouse monoclonal anti-Cx26 and rabbit policlonal anti-Cx26) showing a strong PLA positive signal (red, middle panel). Negative controls, B) non transfected HeLa cells, PLA assay was done using monoclonal anti-Cx26 and policlonal rabbit anti-Cx30, showing no signal (middle panel); C) a stable cell line of HeLa expressing Cx26 transfected with the non compatible Cx43 (with GFP tag, green). No signal was observed in this condition (middle panel), which is in accordance with the literature. Scale bar, 10 μm.

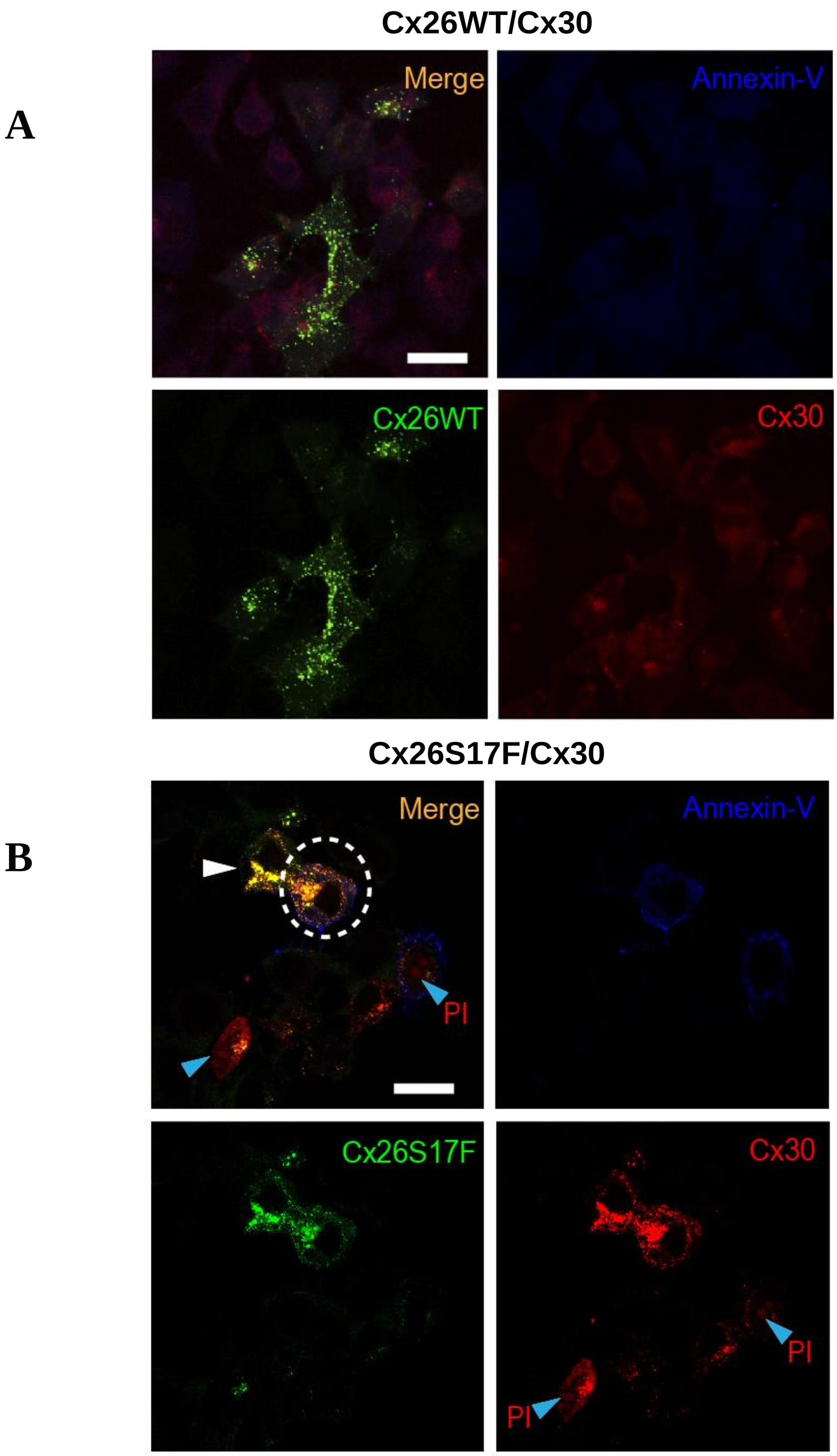

Supplementary Figure S9. Co-expression of Cx26S17F/Cx30 but not Cx26WT/Cx30 increases cell damage and cell death in HeLa cells. Positive signal of annexin V at the membrane and PI at the nucleus of cells co-transfected with Cx30 and Cx26S17F (B), but not with Cx26WT (A). Dotted ellipse (B) demarks a co-transfected cell with annexin V at the membrane, while the cell demarked with the white arrowhead is also co-expressing the two Cxs but it is in good shape. Two cells at the bottom (B), demarked with a cyan arrowhead shows PI positive signal and co-expression of Cx26S17F and Cx30, and rounded morphology as observed in dead cells. No incidence of PI positive signal was detected in cell co-transfected with Cx26WT and Cx30.

Table 1. Correlation index Pearson values, SEM and p value of Cx26WT/Cx30 and Cx26S17F/Cx30 per subcellular area

Cx26WT/Cx30 vs Cx26S17F/Cx30	Mean Cx26WT/Cx30 ± SEM	Mean Cx26S17F/Cx30 ± SEM	p value
Mb	0.75 ± 0.07 N=7	0.69 ± 0.06 N=3	0.5727
Intracellular	0.64 ± 0.07 N=8	0.69 ± 0.05 N=8	0.5768
GJ	0.69 ± 0.06 N=8	0.78 ± 0.07 N=2	0.4249

Table 2. Correlation index Manders 1 (Cx26WT or Cx26S17F, green; over Cx30, red), SEM and p value of Cx26WT/Cx30 and Cx26S17F/Cx30 per subcellular area

Cx26 WT/Cx30 vs Cx26S17F/Cx30	Mean Cx26WT/Cx30±SEM	Mean Cx26S17F/Cx30±SEM	p value
Mb	1.00 ± 0.0 N=7	0.97 ± 0.03 N=4	0.391
Intracellular	1.00 ± 0.0 N=8	0.97 ± 0.01 N=8	0.043
GJ	1.00 ± 0.0 N=8	0.98 ± 0.02 N=2	0.5

Table 3. Correlation index Manders 2 (Cx30, red, over Cx26WT or Cx26S17F, green), SEM and p value of Cx26WT/Cx30 and Cx26S17F/Cx30 per subcellular area

Cx26 WT/Cx30 vs Cx26S17F/Cx30	Mean Cx26WT/Cx30±SEM	Mean Cx26S17F/Cx30±SEM	p value
Mb	0.92 ± 0.02 N=7	0.99 ± 0.002 N=4	0.0134
Intracellular	0.99 ± 0.0006 N=8	0.99 ± 0.0006 N=8	0.0204
GJ	0.98 ± 0.01 N=8	0.99 ± 0.0001 N=2	0.1626

Table 4. Intensities of homomeric Cx26WT, Cx30 or Cx26S17F expression in HeLa cells per subcellular area (Mean ±SEM, N, p value)

		Mean Cx26WT±SEM	Mean Cx30±SEM	Mean Cx26S17F±SEM	p value
Mb	Cx26 WT vs Cx30	3,28 ± 0,79 N=23	3,84 ± 1,26 N=21		0.713
	Cx26 WT vs Cx26S17F	3,29 ± 0,79 N=23		6,82 ± 1,99 N=14	0.1181
	Cx30 vs Cx26S17F		3,84 ± 1,26 N=21	6,82 ± 1,99 N=14	0.2196
Intra	Cx26 WT vs Cx30	48,11 ± 3,04 N=23	30,39 ± 3,8 N=21		0.0008
	Cx26 WT vs Cx26S17F	48,11 ± 3,04 N=23		87,54 ± 2,76 N=14	< 0,0001
	Cx30 vs Cx26S17F		30,39 ± 3,80 N=21	87,54 ± 2,76 N=14	< 0,0001
GJ	Cx26 WT vs Cx30	48,60 ± 3,44 N=23	65,77 ± 3,95 N=21		0.0021
	Cx26 WT vs Cx26S17F	48,60 ± 3,44 N=23		5,64 ± 1,82 N=14	< 0,0001
	Cx30 vs Cx26S17F		65,77 ± 3,95 N=21	5,64 ± 1,82 N=14	< 0,0001

Table 5. Intensities Cx26WT and Cx30 in heteromeric configuration in HeLa cells per subcellular area (Mean ±SEM, N, p value)

	Cx26WT/Cx30						
	Cx26WT			Cx30			
	Mean	SEM	N	Mean	SEM	N	p value
Membrane	14.59	3.25	20	19.35	6.07	19	0.4879
Intracellular	42.46	5.85	19	30.99	6.50	17	0.1970
Gap junction	40.07	6.65	20	42.69	8.45	19	0.8075

Table 6. Intensities Cx26S17F and Cx30 in heteromeric configuration in HeLa cells per subcellular area (Mean ±SEM, N, p value)

	Cx26S17F/Cx30						
	Cx26S17F			Cx30			
	Mean	SEM	N	Mean	SEM	N	p value
Membrane	18.41	6.93	18	20.21	6.09	18	0.8462
Intracellular	60.06	8.58	18	59.78	7.88	18	0.9807
Gap junctior	21.53	7.72	18	20.01	6.47	18	0.8809

Table 7. Relative intensities of Cx30 when interacting with Cx26WT or Cx26S17F in HeLa cells per subcellular area (Mean ±SEM, N, p value)

	with Cx26WT		with Cx26S17F		Cx30
	Mean	SEM	Mean	SEM	p value
Membrane	19.35	6.07	20.21	6.09	0.92
Intracellular	30.99	6.50	59.78	7.88	0.008 *
Gap junctior	42.69	8.45	20.01	6.47	0.04

Table 8. Percentaje of GJ coverage of the apposition zone in HeLa cells expressing homomeric Cx26WT, Cx30 and heteromeric Cx26WT/Cx30 and Cx26S17F/Cx30 (Mean ±SEM, N, p value)

	Mean ± SEM	N	p value
Cx26WT	59.04 ± 2.97	53	0.3823
Cx30	63.64 ± 4.53	29	
Cx26WT/Cx30	48.65 ± 5.49	13	0.0022
Cx26S17F/Cx30	24.33 ± 4.28	12	

Table 9. FRAP recovery rates comparison of homomeric Cx26WT v/s Cx30, and heteromeric Cx26WT/Cx30 v/s Cx26S17F/Cx30 (Mean, SEM and p value)

	Mean ± SEM	N	p value
Cx26WT	0.42 ± 0.08	13	0.9564
Cx30	0.43 ± 0.03	4	
Cx26WT/Cx30	0.47 ± 0.06	8	0.0003
Cx26S17F/Cx30	0.1 ± 0.01	9	
